# Expanding the Role of Sub-Exploited DOE-High Energy Extraction and Metabolomic Profiling towards Agro-Byproduct Valorization: The Case of Carotenoid-Rich Apricot Pulp

**DOI:** 10.3390/molecules25112702

**Published:** 2020-06-11

**Authors:** Thalia Tsiaka, Charalambos Fotakis, Dimitra Z. Lantzouraki, Konstantinos Tsiantas, Eleni Siapi, Vassilia J. Sinanoglou, Panagiotis Zoumpoulakis

**Affiliations:** 1 Institute of Chemical Biology, National Hellenic Research Foundation, 48, Vas. Constantinou Ave., 11635 Athens, Greece; thtsiaka@eie.gr (T.T.); bfotakis@yahoo.com (C.F.); dlantzouraki@eie.gr (D.Z.L.); esiapi@eie.gr (E.S.); 2 Laboratory of Chemistry, Analysis & Design of Food Processes, Department of Food Science and Technology, University of West Attica, Ag. Spyridonos, 12243 Egaleo, Greece; kostastsiant@hotmail.gr

**Keywords:** apricot byproduct, carotenoids, ultrasound-assisted extraction (UAE), microwave-assisted extraction (MAE), nuclear magnetic resonance (NMR) spectroscopy, multivariate chemometric analysis

## Abstract

Traditional extraction remains the method-of-choice for phytochemical analyses. However, the absence of an integrated analytical platform, focusing on customized, validated extraction steps, generates tendentious and non-reproducible data regarding the phytochemical profile. Such a platform would also support the exploration and exploitation of plant byproducts, which are a valuable source of bioactive metabolites. This study deals with the incorporation of (a) the currently sub-exploited high energy extraction methods (ultrasound (UAE)- and microwave-assisted extraction (MAE)), (b) experimental design (DOE), and (c) metabolomics, in an integrated analytical platform for the extensive study of plant metabolomics and phytochemical profiling. The recovery of carotenoids from apricot by-products (pulp) is examined as a case study. MAE, using ethanol as solvent, achieved higher carotenoid yields compared to UAE, where 1:1 chloroform-methanol was employed, and classic extraction. Nuclear magnetic resonance (NMR)-based metabolomic profiling classified extracts according to the variations in co-extractives in relation to the extraction conditions. Extracts with a lower carotenoid content contained branched-chain amino acids as co-extractives. Medium carotenoid content extracts contained choline, unsaturated fatty acids, and sugar alcohols, while the highest carotenoid extracts were also rich in sugars. Overall, the proposed pipeline can provide different the phytochemical fractions of bioactive compounds according to the needs of different industrial sectors (cosmetics, nutraceuticals, etc.).

## 1. Introduction

Plant metabolomics is the answer to traditional phytochemical approaches, which are focused on the analysis of specific targeted metabolites, usually a group of bioactive compounds (i.e., carotenoids, polyphenols, alkaloids, and amino acids), and not on the complete and detailed metabolic profile of the plant substrate or plant byproducts. However, the different composition of the plant matrix, any possible enzymatic degradation or chemical breakdown of plant metabolites, and the lack of a tailor-made validated extraction step crucially affect the final quality of the metabolomic study and the number of identified metabolites [1]. Among these factors, the step of sample preparation is of the utmost importance, since the information provided by high throughput analytical techniques is highly dependent on the selected extraction method. Therefore, special attention should be paid to the development of a comprehensive extraction methodology that can investigate the plant metabolome as exhaustively as possible [1].

Classic extraction is the method-of-choice in the majority of analytical studies [2,3]. Most of the time, traditional extraction methods are based on the previous experience and knowledge of the researchers, who apply them without further optimization or validation. Meanwhile, high energy extraction methods (e.g., ultrasound-assisted extraction (UAE) or microwave-assisted extraction (MAE)) are gaining ground, but are still ignored or sub-exploited in plant metabolomics studies. Nevertheless, these techniques circumvent decisive bottlenecks of classic approaches related to the extraction cost, efficiency, fractionation ability, and time [4]. In addition, the optimization of high energy extraction through experimental design (DOE) models provides answers to questions such as (a) which parameters are crucial for the extraction?, (b) do the interactions between extraction parameters critically affect extraction yields?, and (c) is it possible to enrich the obtained extract with different co-extracting phytonutrients from the substrate by modifying and adjusting the extraction conditions?. The latter issue is of unique importance for the industry of natural products since, even when an extraction is selective, the final natural extract also contains other secondary metabolites in lower concentrations [5,6]. Therefore, the upgraded role of DOE-optimized-high energy extraction in plant metabolomics merits further investigation to generate a robust sample preparation step for further analytical studies [7].

Recently, European Union reports revealed that almost 70% of total food processing ends up as waste or byproducts and foresaw an increase of 30% in the disposal of food byproducts by 2020 [8]. This insight, along with the intensive use of plant-derived natural ingredients, the threat of wild crafting, unsustainable harvesting practices, undernourishment, and increased food prices [9], forged the concepts of ‘food from food’ and ‘byproducts to co-products’ through the use of low-waste agro-industry production and the development of modern pipelines for the extraction and valorization of therapeutic natural agent/extracts from plant byproducts [10,11,12].

Reports by the Food and Agriculture Organization Corporate Statistical Database (FAOSTAT) place Greece in the top twenty producer and exporting countries of apricots (*Prunus armeniaca*) worldwide. As a sequent, a large production size generates immense quantities of apricot byproducts with high contents of carotenoids and sugars, and then secondarily, phenolics (rutin, catechin, epicatechin, and chlorogenic acid) and amino acids [13]. Focusing on apricot pulp carotenoids, the orange color unveils the presence of carotenes (α-, β-, and γ- carotene, etc.) rather than xanthophylls (zeaxanthin, lutein, β-cryptoxanthin, etc.). Among α- and β-carotene, the β-isomer is the prevailing form in plant tissues, while zeaxanthin is the main xanthophyll, but its content can be 50-times lower than that of β-carotene [14]. Therefore, apricot byproducts may be a low-cost and sustainable natural source of potential bioactive compounds [13].

In particular, β-carotene acts as an antioxidant and major precursor of vitamin A (retinol), since it presents 100% provitamin A activity due to its two β-ionone rings [13]. It enhances the immune system by regulating intercellular signaling pathways, cell differentiation, growth factors, and cell apoptosis. Moreover, it offers protection against atherosclerosis and coronary diseases [15]. In addition, there is strong evidence for the significance of β-carotene metabolic pathways in the risk reduction for some types of cancer (lung, head, prostate, skin, liver, breast, and colorectum cancer). Nonetheless, in order to avoid negative effects, many clinical intervention studies recommend low-dosage dietary intake levels of β-carotene supplements [16]. Cases of multi-carotenoid supplementation indicated positive results for Alzheimer diseases and vision impairments by balancing the adverse effects of different kinds of degeneration, UV radiation, malfunctions, and oxidative stress [13].

Considering the importance of natural extracts as a novel and promising nutraceutical and cosmeceutical trend, the current work aims to provide an integrated platform for the fast and reproducible delivery of extracts highly enriched in certain phytochemicals. The production and study of carotenoid-rich extracts from apricot’s industrial by-products is selected as a case study. To fulfill this aim, the individual objectives of the study are (i) to access information not only for the targeted substances (carotenoids), but also for important untargeted secondary metabolites; (ii) to unveil the metabolite variations in carotenoid-rich high-energy extracts; and (iii) to classify extracts based on the extraction conditions. 

## 2. Results and Discussion

### 2.1. Extraction Solvent

Selecting the ideal extraction solvent for a certain class of bioactive compounds is based on the (a) substrate nature, (b) target compound’s physicochemical properties, (c) solvent’s polarity/affinity to the extracted analytes, and (d) extraction technique principals/mechanisms [17].

**Figure 1:**
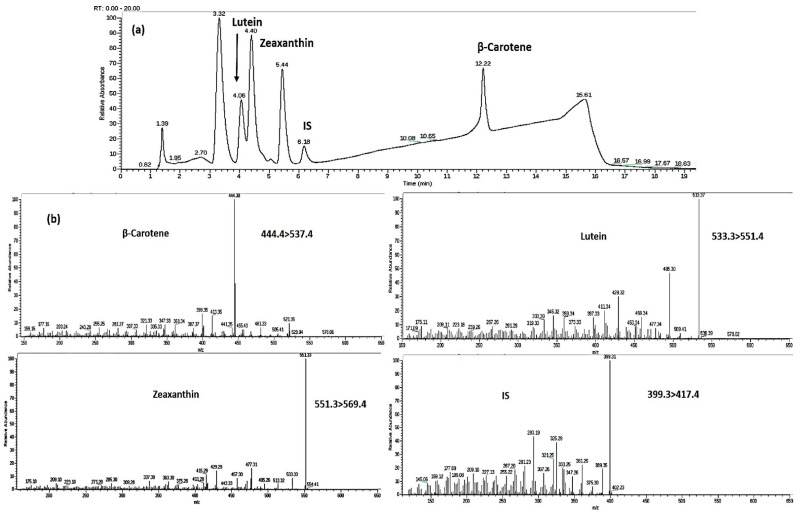
Carotenoids’ (**a**) chromatographic peaks and (**b**) mass transitions.

Three *trans*-carotenoids—β-carotene, lutein, and zeaxanthin—were detected and quantified in apricot pulp. Carotenoids’ peaks and mass transitions are shown in Figure 1a,b.

**Figure 2:**
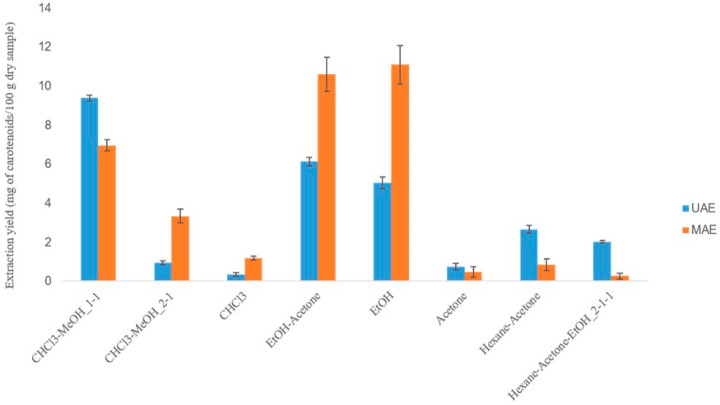
Selecting the optimal ultrasound-assisted extraction (UAE) and microwave-assisted extraction (MAE) solvent system for carotenoid extraction from apricot pulp.

Based on the lipophilic nature of carotenoids, eight different extraction systems were investigated (Table S1) and selected based on (i) the established knowledge regarding the solvents used for carotenoid extraction; (ii) the physicochemical properties of the solvents, which affect and differentiate their extracting ability with UAE or MAE; and (iii) the results of previous studies of our lab concerning the extraction of carotenoids from other substrates [14]. According to the results (Figure 2), alcohols and most of their mixtures provided higher extraction yields in both techniques.

The extracting ability of UAE relies on the acoustic cavitation phenomenon, which is induced by solvents with a high surface tension, low viscosity, and vapor pressure. All examined solvents exhibited similar values of surface tension, but methanol and ethanol had a significantly lower vapor pressure compared to the other extracting agents. In terms of the two alcohols, methanol has a lower viscosity than ethanol and therefore, its combination with a solvent which also has a low viscosity (chloroform) provided higher extraction yields [18]. The dielectric constant values and transparency to microwaves (MWs ) determine the adequacy of an MAE solvent. Since the MAE mechanism is a result of dipole rotation and ionic conductivity, solvents with a high dielectric constant (usually more polar solvents) efficiently absorb MWs and become more suitable for MAE. Therefore, ethanol emerged as the optimal solvent for carotenoid MAE [19].

### 2.2. Extraction Temperature

The extraction process using microwaves and ultrasounds involves a complex interplay between different phenomena which contribute to solid matrix disruption and solvent diffusion into the substrate. The temperature requires special attention during the extraction process due to its binary effect on the extracted compounds. On the one hand, increased temperatures enhance cell disruption and the release of target compounds to the solvent medium, while at the same time, a rampant increase of temperature may result in the degradation of extracted constituents [18]. In our case, the UAE temperature was adjusted to 30–35 °C by inserting the extraction flask into an ice bath and monitoring the temperature throughout the extraction procedure. Low temperatures are recommended for UAE, in order to avoid an increase of the solvent’s vapor pressure, which limits the collapse of cavitation bubbles and, by extension, the appearance of sonochemical effects [17]. In open vessel MAE, the extraction temperature is interrelated with the applied MW energy and extraction time. However, the MAE temperature never exceeds the boiling point of the extraction solvent. Increased temperatures enhance the extracting potency of MAE solvent due to the viscosity/surface tension decrease and cell component disruption [20]. As previously reported [21], β-carotene is stable until 60 °C, so the MAE temperature was set to this value.

### 2.3. UAE and MAE Optimization Using DOE Models

The effect of the extraction time (X_1_, minutes), US/MW power (X_2_, W), and solvent/material ratio (X_3_, mL g^−1^) on the carotenoid extraction yield was evaluated using 2^3^ full factorial and Box–Behnken (BBD) design. All experimental runs were performed using a 1:1 *v*/*v* mixture of methanol-chloroform, for UAE and ethanol, for MAE.

#### 2.3.1. Screening Design (2^3^ Full Factorial Design)

A screening design was applied in order to (i) limit the wide values’ range of the selected factors in a region where higher yields were achieved and (ii) direct the imminent optimization model (response surface model) to this region. The eight experiments of the 2^3^ full factorial design were carried out in a random order to avoid systematic errors. The extraction yields of 2^3^ design experiments are shown in Table S2a,b. The goodness-of-fit for the screening models was assessed using the determination coefficient (R^2^) and the determination coefficient adjusted for the degrees of freedom (R^2^_adj_). The first index indicates how well the produced models fit the dataset and the second one determines which terms of the equation, proposed by the models, truly affect the response. A model where the values of the two coefficients are higher than 0.8 and their difference is around 0.2 describes the dataset well. Regarding our case, the 2^3^ design could reliably direct the upcoming Box–Behnken model to the value range where higher carotenoid yields would be obtained, since R^2^ = 0.920 and R^2^_adj_ = 0.814 for UAE and R^2^ = 0.987 and R^2^_adj_ = 0.954 for MAE. Based on the *p*-values, special attention should be paid to the adjustment of the solvent/material ratio (*p*-values ≤ 0.05) in both extraction techniques (Table S3a). According to two-dimensional (2D) contour plots, UAE performed better at extraction times over 10 min when the ultrasound (US) power and solvent/material ratio were set at high values (≥600 W and ≥30 mL g^−1^, respectively) (Figure S1a–c). An MAE optimization model should be focused on extraction times from 5 to 20 min, an MW power lower than 150 W, and a high solvent/material ratio (≥50 mL g^−1^) (Figure S1d–f).

#### 2.3.2. Response Surface Methodology Models (Box–Behnken Design)

Box–Behnken optimization models, driven by the trends revealed in the prior screening designs, were applied for accomplishing the optimal extraction values of high energy techniques. The model-proposed experimental runs and carotenoid content for each run are illustrated in Table S2a,b. The processing of the dataset resulted in two (one for each extraction method) predictive second-order polynomial equations. Terms with high *p*-values (*p*-value ≥ 0.05) were considered statistically insignificant and excluded from the equations. Therefore, the final model equations (Equations (1) and (2)), expressed in normalized values, consisted of the following terms:

UAE yield (mg of total carotenoids 100 g^−1^ dry sample) = 8.706 − 1.80x_1_ + 1.003x_1_^2^ + 0.80x_2_^2^ + 1.021x_3_^2^ + 0.5280x_1_^2^x_2_ + 0.4674 x_1_x_2_^2^ − 1.761x_1_x_3_ − 1.819x_1_^2^x_3_ + 2.023x_2_x_3_, (1)

MAE yield (mg of total carotenoids 100 g^−1^ dry sample) = 15.91 − 0.3208x_1_^2^ + 2.767x_2_ − 1.116x_2_^2^ + 1.372x_1_x_2_ + 1.554x_1_x_2_^2^ − 2.332x_1_x_3_ − 3.784x_2_x_3_. (2)

The significance of all equation terms is illustrated in Pareto charts (Figure S2), where the important terms are the ones that outstrip the threshold of *p*-value ≤ 0.05 (red line). Taking into consideration the *p*-value ≤ 0.05 criterion presented in the ANOVA table (Table S3b), UAE was significantly affected by the linear term of the extraction time (x_1_), the quadratic term of the solvent/material ratio (x_3_^2^), and the interaction between the solvent/material linear term and (a) the extraction time quadratic term (x_1_^2^x_3_) and (b) the US power linear term (x_2_x_3_) (Figure S2a). UAE yields presented a directly proportional linear relationship with the extraction time and a directly proportional exponential relationship with the solvent/material ratio, explained by the positive sign of x_1_ and x_3_^2^ terms in Equation (1). Furthermore, the linear (x_2_) and quadratic term (x_2_^2^) of MW power, the interaction between the extraction time linear term and MW power quadratic term (x_1_x_2_^2^), and the interaction between the solvent/material linear term and the linear terms of the extraction time (x_1_x_3_) and MW power (x_2_x_3_) played the most important roles in MAE carotenoid yields. As illustrated in Figure S2b, the linear term of MW power affected the final result more than the quadratic term. Therefore, the positive sign of the x_2_ term in Equation (2) revealed the directly proportional dependence of MAE yields and MW power.

The BBD models were considered reliable according to R^2^ and R^2^_adj_ values_,_ which were relatively high and close to one another (R^2^ = 0.886 and R^2^_adj_ = 0.714 for UAE and R^2^ = 0.876 and R^2^_adj_ = 0.767 for MAE). Moreover, only a percentage of around 10% of the total variations was not interpreted by the produced models. The good fitness of the BBD models for the experimental data was also established by the *p*-values corresponding to the total model and not each term, which were ≥0.05 (*p*-value UAE = 0.941, *p*-value MAE = 0.979), confirming that there was no models’ lack-of-fit (Table S3b). In addition, the robustness of our models was evaluated through the standard deviations (UAE stdev = 2.2, MAE stdev = 2.6) of the four repetitions at the center points (0,0,0). All previous results are presented in the ANOVA table (Table S3b). 

#### 2.3.3. Evaluate the Effects of Extraction Factors under Optimization

**Figure 3:**
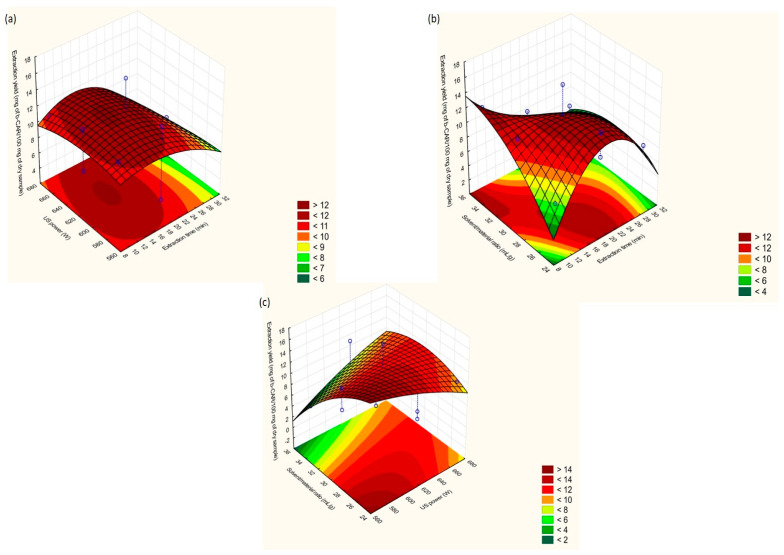
Response surface methodology (RSM) plots for UAE: (**a**) extraction time vs. US power; (**b**) US power vs. solvent/material ratio; (**c**) solvent/material ratio vs. extraction time.

**Figure 4:**
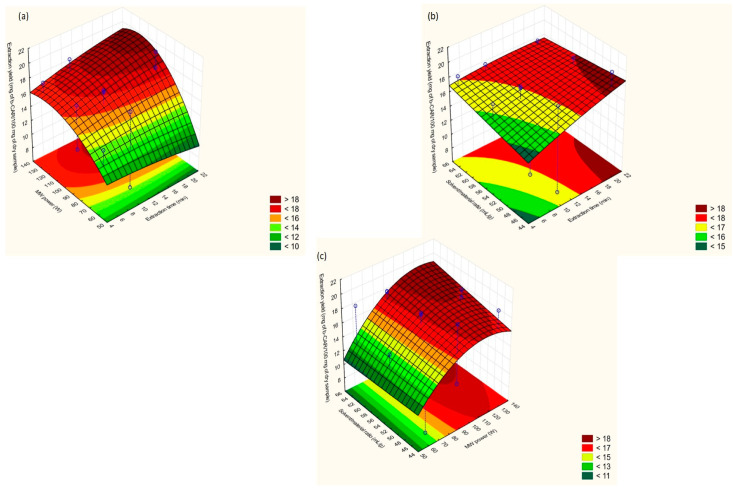
RSM plots for MAE: (**a**) extraction time vs. MW power; (**b**) MW power vs. solvent/material ratio; (**c**) solvent/material ratio vs. extraction time.

Three-dimensional (3D) response surface methodology (RSM) plots were generated for an evaluation of the effects of the DOE-optimized extraction factors on the carotenoid yield when UAE (Figure 3a–c) and MAE (Figure 4a–c) were employed. RSM plots depict the combinatorial effect, each time, of two of the investigated extraction factors on the carotenoid content, while the third parameter is kept constant at the medium value level (0). 

##### Extraction Time

As stated in Figure 3a,b, UAE was favored at extraction times between 10 and 20 min. A closer look at these figures showed that at even a 10 min extraction time, high extraction could be achieved when the US power and solvent/material ratio had values of 600–620 W and 30-35 mL g^−1^, respectively. Studies regarding carotenoid UAE from plant tissues indicated that carotenoids may be degraded at a prolonged extraction time (over 15 min). Therefore, a 10 to 20-min period seems ideal for carotenoid recovery from agro-byproducts [22].

Compared to UAE, MAE is a more complex process due to the interrelation of the extraction time, temperature, and MW power. Other research groups [23] have examined the co-dependence of these three parameters and showed that extended extraction times of over 15 min resulted in high carotenoid yields when the MW power was adjusted to 100–140 W. This was also confirmed in the present work (Figure 4a). In addition, a higher extraction time and higher solvent/material ratio provided higher carotenoid yields (Figure 4b).

##### US/MW Power

In general, the positive impact of sonochemical effects is more pronounced at an increased US power, which improves cell wall disruption and solvent penetration. Recent research has asserted that a US power of over 250 W negatively affected the extraction efficiency, but US exposure lasted for long periods (40–100 min) [17]. However, a high US power (580–620 W) recovered high concentrations of carotenoids when applied for short periods (8–15 min) (Figure 3a,c).

An increasing trend for extraction yields was observed (Figure 4a,c) at 100–140 W of MW power. For this power range, the increase of the extraction temperature was slower and steady throughout the MAE process. Therefore, the release of target compounds from substrate tissues was more efficient due to more gradual solvent heating. A higher MW power may (i) deteriorate the extracted labile molecules or (ii) cause solvent losses from extreme solvent heating and lead to reduced extraction rates. On the other hand, low irradiation values do not cause complete cell disruption and extraction yields are thus usually lower [23].

##### Solvent/Material Ratio

According to the UAE Pareto chart (Figure S2a), the solvent/material ratio was viewed as the most critical extraction factor. As showcased in Figure 3b,c, solvent volumes between 25 and 35 mL seem to be high enough to adequately diffuse and dissolve the extracted compounds. The addition of extra volume did not maximize carotenoid migration to the solvent due to the increase of the diffusion distance from the extracting medium and the examined matrix [24].

The MAE regression equation (Equation (2)) indicated, in quite significant terms, the interaction between the solvent/material ratio and MW power and extraction time (Figure S2b). When the extraction time varies from 15 to 20 min and MW power from 100 to 120 W, the solvent/material ratio should be adjusted to 44–56 (mL g^−1^) for obtaining the maximum extraction yields (Figure 4b,c). Larger solvent volumes demanded longer periods (≥20 min) of MW irradiation at the mentioned values, in order to achieve uniform solvent heating and thus efficient carotenoid recovery. Nevertheless, extended extraction times of MW radiation could promote the degradation of thermosensitive compounds due to excessive heating of the extraction solvent, such as β-carotene or zeaxanthin [25].

##### Optimal Extraction Conditions

The third and final step of DOE optimization strategies refers to the conduction of experiments around the regions of the values where the response is maximized according to the equations produced by BBD (Equations (1) and (2)). Three experimental combinations were proposed and performed optimally. The lack of a significant difference (Student’s t-test) between the predicted and experimental values proved the reliability of DOE-optimized extractions (Table S4).

The optimal values of UAE and MAE parameters for carotenoid recovery from apricot byproducts are presented in Table 1.

### 2.4. Comparison of Optimized UAE, MAE, and Conventional (Folch) Extractions

At this point, in order to prove the efficiency of MAE and UAE, it is critical to provide comparative data for MAE and UAE and Folch, which is a widely used conventional extraction methodology for the recovery of lipid components, such as carotenoids. According to the results delivered by LC-MS/MS analysis for the carotenoid content of apricot pulp, β-carotene, zeaxanthin, and lutein were quantified (Table S2).

MAE extracts almost 2.5-times more β-carotene than UAE. This outcome is probably related to (a) the high polarity of ethanol, which allows the absorbance of MW energy and the acceleration of the MAE process, and (b) the combinatorial effect of MW power and the increased (compared to UAE) extraction temperature, which causes looseness and disruption of the tight cell structure and the enhanced diffusion of β-carotene in ethanol [21]. Additionally, the Folch method does not appear to be an apt choice for β-carotene recovery from fruit tissues, since the β-carotene content is 19-fold and 7.5-fold lower than in MAE and UAE, respectively.

The lower extraction yield of UAE compared to MAE may be attributed to the degradation of β-carotene to *cis*-isomers, oxygenated derivatives, and β-*apo*-carotenals (i.e., 15-*Z*-β-carotene, di-*Z*-β-carotene, and 9-*cis*-β-carotene) due to isomerization, oxidation, and cleavage reactions caused by US [26].

The same trend was also observed for apricot’s xanthophylls. The Folch method recovered a minor amount of zeaxanthin and lutein, which was one order of magnitude lower than the xanthophylls’ content of high energy techniques. Unlike the significant difference in the β-carotene concentration, UAE and MAE extracted equal amounts of zeaxanthin and lutein (Table 2). The ratio of *trans*-xanthophylls to *trans*-β-carotene in MAE and Folch is around 1:20–1:25, while in UAE, this ratio is 1:10, implying that the degradation of β-carotene in UAE was more severe (Table 2).

The content of carotenoid-rich fruits and fruit byproducts, like apricots, varies markedly when the variety, cultivar and hybrids, geographical origin, climatic differences, genotype, ripening, and development stages alter. Hence, it is a quite intricate task to deliver an unbiased comparison of the different extraction techniques used to extract carotenoids from such substrates. Nevertheless, the current MAE process managed to extract four-times more β-carotene, but similar xanthophyll equivalents, when compared to accelerated solvent extraction (ASE), while UAE and ASE resulted in almost the same final carotenoid content [27]. Classic extraction, applied to different varieties of apricots cultivated in New Zealand and USA, achieved a β-carotene yield close to that of MAE. The higher yields of lutein obtained by Leong et al.’s (2012) classic method may support the hypothesis of xanthophyll’s isomerization under certain conditions of high energy extractions [14,28]. To wrap up the results of the extraction of carotenoids using high energy techniques, it becomes clear that especially MAE’s role should be revised and upgraded.

### 2.5. NMR-Based Metabolic Profiling for DOE Apricot Extracts to Elucidate Co-Extractives

Nuclear magnetic resonance (NMR) is one of the most implemented and efficient analytical platforms used for the elucidation of metabolites from complex mixtures, including natural extracts. Therefore, NMR spectroscopy was integrated as an additional complementary technique for the simultaneous elucidation of the high concentration co-extracted metabolites other than carotenoids that shape the different metabolic profiles of each group [29] (Figure S3). The examined samples were (i) the extracts of all of the different UAE and MAE solvents; (ii) indicative DOE-high energy extracts with a lower, medium, and higher carotenoid content; (iii) the optimal UAE and MAE extracts; and (iv) Folch extracts. Table 3 presents the assignment of 15 major metabolites identified in the ^1^H-NMR spectra of the different samples. For the assignment, previous literature findings and 2D NMR experiments (gCOSY, gHSQC, and gHMBC) were utilized (Figure S4). 

**Figure 5:**
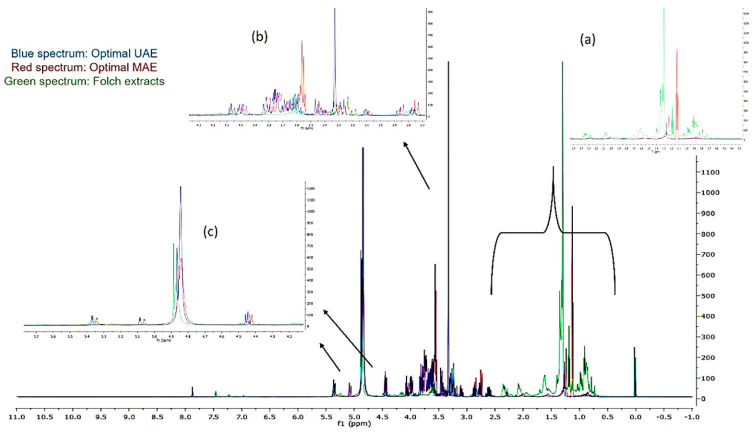
Regions of (**a**) amino acids, (**b**) fatty acids and myo-inositol, and (**c**) mono- and di-saccharides in superimposed ^1^H-NMR spectra of optimal UAE, optimal MAE, and Folch extracts.

In Figure 5, the superimposed spectra belong to the (a) optimal UAE (blue spectrum) (b) optimal MAE (red spectrum), and (c) Folch apricot pulp extracts (green spectrum). Folch extracts presented peaks in the amino acid region (Figure 5a). On the other hand, the optimal UAE and optimal MAE extracts contained, according to Figure 5b,c, fatty acids and myo-inositol or sugars, respectively.

**Figure 6:**
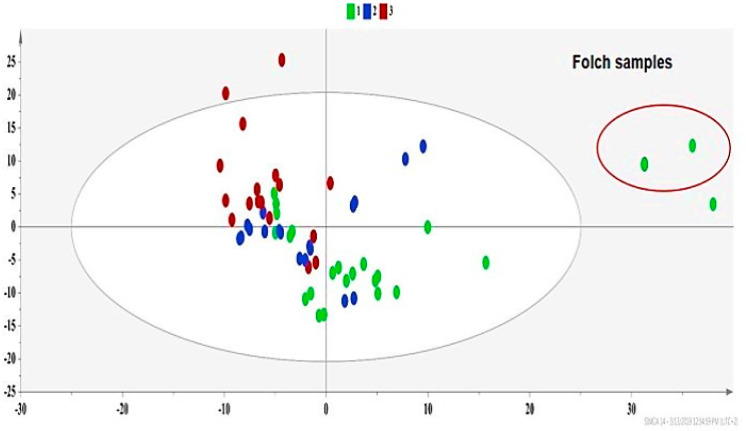
PCA of experimental design (DOE)-extracts PCA-X, where A = 2, N = 57, R^2^X(cum) = 0.73, Q^2^(cum) = 0.57, green dots = apricot pulp samples with a low extraction yield (≤5 mg carotenoids 100 g ^−1^ dry sample), blue dots = apricot pulp samples with a medium extraction yield (5–15 mg carotenoids 100 g^−1^ dry sample), and red dots = apricot pulp samples with a high extraction yield (≥15 mg carotenoids 100 g^−1^ dry sample).

Principal component analysis (PCA) provided an overview of the trends and possible outliers of high energy extracts [30]. This unsupervised analysis copes with how the extraction parameters shape the profile of co-extracted metabolites. The PCA model (Figure 6) framed three groups. Group 1 (green dots) contained extracts with a relatively low carotenoid content (under 5 mg 100 g^−1^ dry sample), Group 2 (blue dots) contained those with a medium carotenoid content (between 5–15 mg 100 g^−1^ dry sample), and Group 3 (red dots) consisted of extracts with a high carotenoid yield (over 15 mg 100 g^−1^ dry sample) (Figure 6 and Table S5).

The primary factor for sample classification was the polarity of the extraction solvent, rather than the extraction technique used, since Group 1 (green dot), which shows a different trend from Group 3 (red dots), mostly contains extracts of less polar solvents, regardless of the extraction technique used (Figure 6 and Table S5). The effect of the extraction method was outlined implicitly, since every solvent shows a different behavior when interacting with US or MW due to its different physical properties (polarity, viscosity, vapor pressure, diffusion coefficient, etc.).

In Figure 6, Folch samples are outliers, proving that the conventional technique exhibits a distinct metabolic profile compared to high energy approaches. This fact is in accordance with the LC-MS/MS results (Table 2), where classic methods also presented lower carotenoid yields. Folch samples exhibited a high content of valine, isoleucine and leucine, fatty acids, lactic acid, alanine, and malic acid, as presented in the contribution plot in Figure S5.

**Figure 7:**
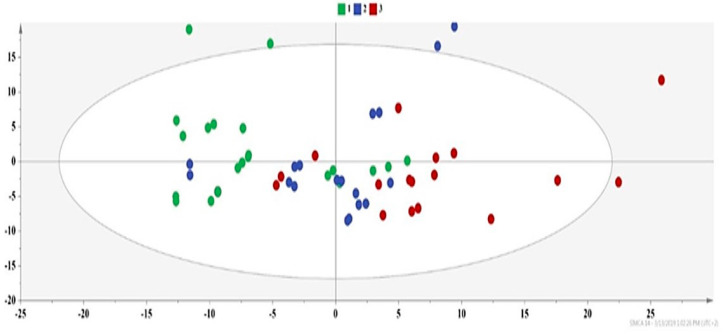
PCA of DOE-extracts excluding Folch samples PCA-X, where A = 2, N = 54, R^2^X(cum) = 0.87, Q^2^(cum) = 0.67, green dots = apricot pulp samples with a low extraction yield (≤5 mg carotenoids 100 g^−1^ dry sample), blue dots = apricot pulp samples with a medium extraction yield (5–15 mg carotenoids 100 g^−1^ dry sample), and red dots = apricot pulp samples with a high extraction yield (≥15 mg carotenoids 100 g^−1^ dry sample).

We excluded the Folch samples and produced a second PCA model (Figure 7), where the trend for the formation of three groups is still evident. Besides the solvent polarity, the grouping of extracts (Figure 7) gave a hint regarding the effect of extraction parameters, highlighting the US/MW power and the solvent/material ratio as more critical variables. More explicitly, Group 2 mainly includes extracts of more polar solvents, such as methanol and ethanol, and UAE extracts with a higher US power and solvent/material ratio (Tables S2a and S5). Furthermore, the common factors between the extracts of Group 3, which are primarily MAE extracts, are a higher MW power and solvent/material ratio (Tables S2b and S5). 

**Figure 8:**
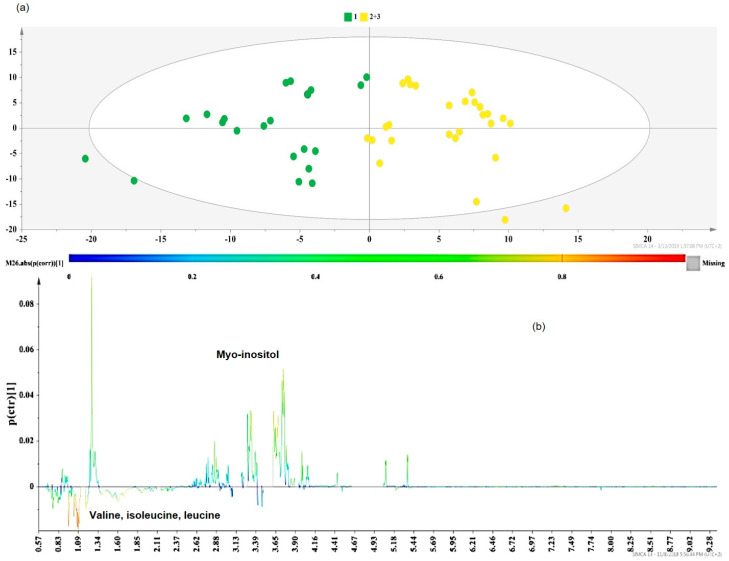
(**a**) Orthogonal Projections to Latent Structures Discriminant Analysis (OPLS-DA) model of Group 1 vs. Group 2+3 DOE apricot extracts, Pareto scaled, where A = 1 + 1 + 0 (N = 48, R^2^Y(cum) = 0.706, Q^2^(cum) = 0.596, *p* < 0.05), green dots = apricot pulp samples with a low extraction yield (≤5 mg carotenoids 100 g^−1^ dry sample), and yellow dots = apricot pulp samples with a higher extraction yield (between 5 and more than 20 mg carotenoids 100 g^−1^ dry sample); (**b**) S-line plot of the OPLS-DA model.

**Figure 9:**
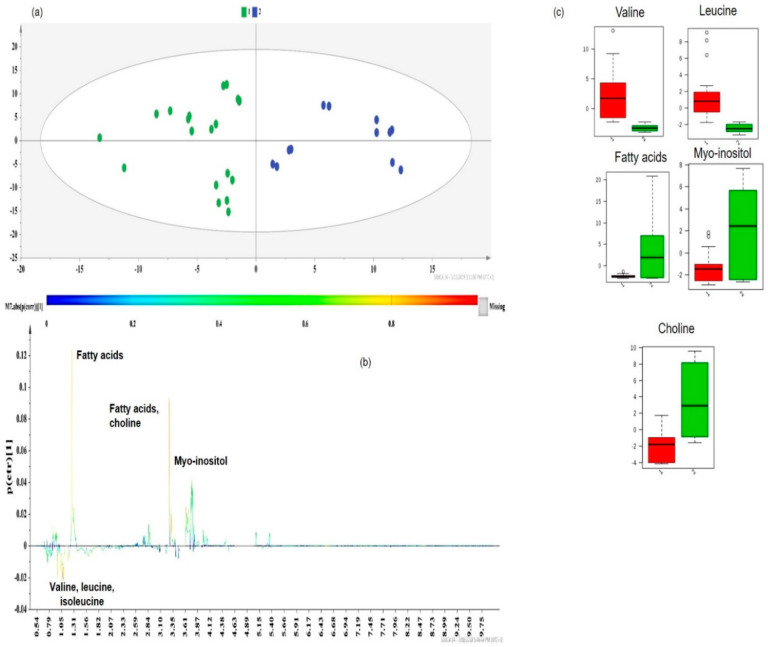
(**a**) OPLS-DA model of Group 1 vs. Group 2 DOE apricot extracts, Pareto scaled, where A = 1 + 1 + 0 (N = 31, R^2^Y(cum) = 0.725, Q^2^(cum) = 0.630, *p* < 0.05), green dots = apricot pulp samples with a low extraction yield (≤5 mg carotenoids 100 g^−1^ dry sample), and blue dots = apricot pulp samples with a medium extraction yield (5–15 mg carotenoids 100 g^−1^ dry sample); (**b**) S-line plot of the OPLS-DA model; (**c**) box-plots of the discriminant metabolites, where red box-plots correspond to Group 1 (green dots) and green box-plots correspond to Group 2 (blue dots).

**Figure 10:**
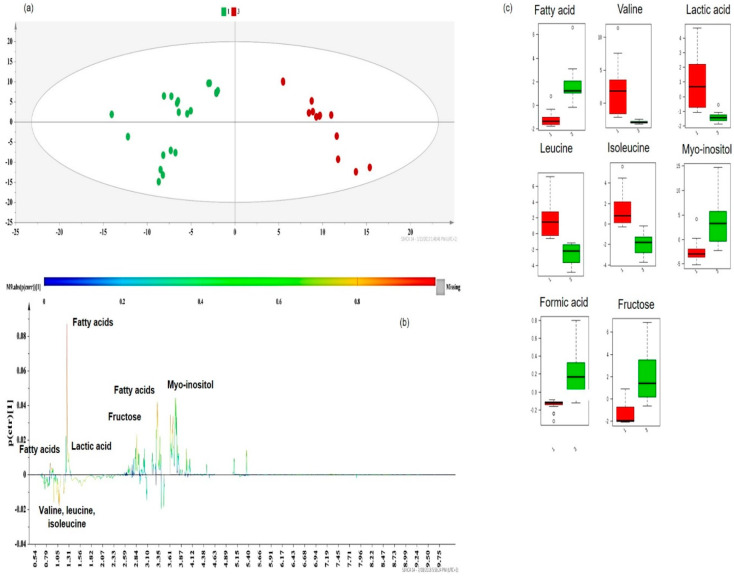
(**a**) OPLS-DA model of Group 1 vs. Group 3 DOE apricot extracts, Pareto scaled, where A = 1 + 1 + 0 (N = 32, R^2^Y(cum) = 0.889, Q^2^(cum) = 0.836, *p* < 0.05), green dots = apricot pulp samples with a low extraction yield (≤5 mg carotenoids 100 g^−1^ dry sample), and red dots = apricot pulp samples with a medium extraction yield (≥15 mg carotenoids 100 g^−1^ dry sample); (**b**) S-line plot of the OPLS-DA model; (**c**) box-plots of the discriminant metabolites, where red box-plots correspond to Group 1 (green dots) and green box-plots correspond to Group 3 (red dots).

**Figure 11:**
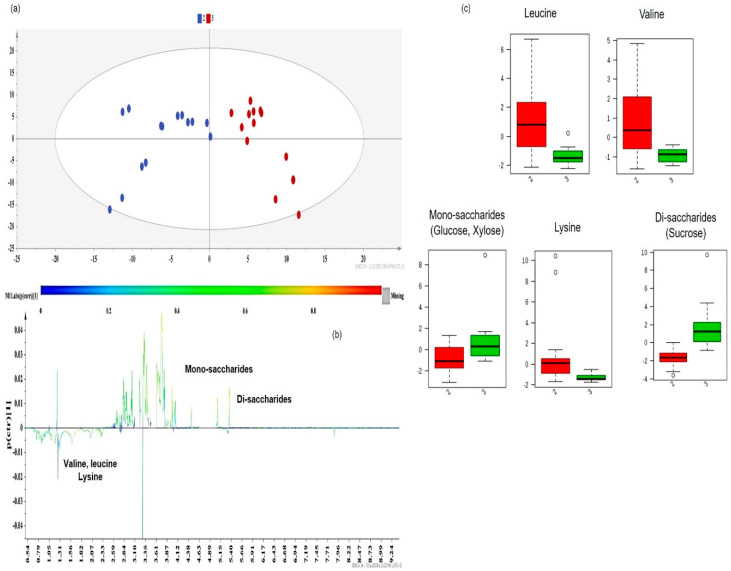
(**a**) OPLS-DA model of Group 2 vs. Group 3 DOE apricot extracts, Pareto scaled, where A = 1 + 1 (N = 27, R^2^Y(cum) = 0.780, Q^2^(cum) = 0.642, *p* < 0.05), blue dots = apricot pulp samples with a low extraction yield (5–15 mg carotenoids 100 g^−1^ dry sample), and red dots = apricot pulp samples with a medium extraction yield (≥15 mg carotenoids 100 g^−1^ dry sample); (**b**) S-line plot of the OPLS-DA model; (**c**) box-plots of the discriminant metabolites, where red box-plots correspond to Group 2 (blue dots) and green box-plots correspond to Group 3 (red dots).

Subsequently, class information was embedded in supervised models of Orthogonal Projections to Latent Structures Discriminant Analysis (OPLS-DA) to identify the key metabolites responsible for extract differentiation [2]. The OPLS-DA models provided a valid separation of DOE-extracts according to their R^2^Y(cum) and Q^2^(cum) values. In each case, the discriminant co-extractives were highlighted by S-line plots (Figure 8b, Figure 9b, Figure 10b and Figure 11b). The discriminant power of each metabolite is demonstrated with a color code and shows an increasing trend from green to red. Therefore, the important metabolites’ peaks for cluster discrimination are depicted with an orange to red color. The respective contents of each metabolite responsible for the discrimination were framed in box-plots (Figure 9c, Figure 10c and Figure 11c), disclosing a pattern in line with the carotenoid yield and UAE/MAE parameters. Permutation testing and receiver operating characteristic (ROC) curves were employed and verified the OPLS-DA model’s reliability (Figure S6a–d).

In particular, the class information enclosed in the first OPLS-DA (Figure 8a) model included the group with a low carotenoid content (Group 1 in green) and one new group comprised of Group 2 and Group 3 (Group 2,3 in yellow), due to the localization of most of Group’s 2 samples together with Group 3 in the PCA model presented in Figure 7.

Extracts with low carotenoid yields (Group 1, green dots) were enriched in branched-chained hydrophobic amino acids (valine, leucine, and isoleucine) (Figure 8b). The above-mentioned amino acids are among apricots’ beneficial secondary components due to their numerous biological activities [31]. The co-extraction of these metabolites was favored in more nonpolar solvents (acetone, chloroform, and *n*-hexane) or their mixtures with methanol. According to previous studies [32], the UAE conditions, especially the time and US power, can initiate amino acid degradation reactions in cases where highly polar solvents are used. Therefore, the combination of less polar solvents with a relatively low US power (575–625 W) (Tables S2a and S5) in Group 1 samples seems to provide apricot pulp extracts with important levels of certain amino acids [32]. 

Myo-inositol was highlighted as an additional marker discriminating Group 1 (low carotenoid content, green dots) from the cluster of Groups 2 and 3 (higher carotenoid yield, yellow dots) (Figure 8b). Myo-inositol is a sugar polyalcohol which participates in plant development by promoting the biosynthesis of molecules responsible for the cell wall structure [33]. Our results are in agreement with previous results, where cyclitols (myo-inositol, d-pinitol, etc.) were mostly extracted at higher UAE solvent volumes and extraction times [34] (Tables S2a and S5) (Groups 2,3 in yellow). As stated in other studies [35], MAE extracts containing inositol were obtained at short extraction times (≤20 min) and high solvent/material ratios, which is a finding that is in accordance with our study (Tables S2b and S5) (Groups 2,3 in yellow).

An in-pairs OPLS-DA investigation of the three carotenoid-dependent groups pinpointed additional differentiating metabolites. Other prevalent components in the separation of Group 1 (low carotenoid yields, green dots) and Group 2 (carotenoid yields between 5 and 15 mg 100 g^−1^ dry sample, blue dots) (Figure 9a) along the second principal component (PC2) were choline and fatty acids, which are key metabolites of Group 2 (Figure 9b). Box-plots of Figure 9c show that in Group 1 (red bars), where less polar solvents were used for extraction (Table S5), hydrophobic amino acids, valine, and leucine presented a higher content. The presence of these amino acids in Group 2 (red dots) was not significant, as the solvent of the samples, which were UAE extracts (Table S5), was more polar (a mixture of methanol and chloroform). However, the use of a unique common solvent in all samples of Group 2 provided more reproducible results regarding the recovery of valine and leucine, since all samples of Group 2 showed a small distribution around the median value (Figure 9c, green bars). 

On the other hand, choline, fatty acids, and myo-inositol characterize the extracts of Group 2. According to box-plots, Group 1 (green dots) contained a very low content of fatty acids (Figure 9c, red bars), since UAE or MAE fatty acid extraction requires a longer extraction time than the one applied (15 min for both techniques) [36,37]. 

The dataset of Group 2 (blue dots) comprises UAE extracts obtained by applying a unique-for-each-sample combination of extraction conditions, since they correspond to different runs of 2^3^ and BBD design (Table S2a). Therefore, the higher variability in the myo-inositol and choline range (Figure 9c, green bars) can be explained. It is worth mentioning that most of the samples of Group 2 were acquired at a higher US power (≥600 W) and solvent/material ratios (≥30 mL g^−1^) (Tables S2a and S5).

Choline is acknowledged as an essential macronutrient, mainly present in lipid foods or plant oils (i.e., apricot kernel oil) as choline derivatives with lipid components (glycerophosphatidylcholine, phosphocholine, and sphingomyelin) [38]. Moreover, oleic (C18:1) and linoleic (C18:2) acids are two of the most predominant fatty acids in the lipid fraction of apricot byproducts, with a content of ~20% and ~10% of the total unsaturated fatty acids, respectively. As shown in Figure 9b, extracts of nonpolar solvents and UAE extracts with a relatively high solvent/material ratio (30–35 mL g^−1^) and US power (625–675 W) belonging in Group 2 (blue dots) (Tables S2a and S5) presented increased levels of choline and fatty acids. Specifically, fatty acid concentration differences and variations between the groups were determined by using the characteristic peak at 1.30 ppm, which corresponds to the overlap of methylene groups (-CH_2_-) (except for those in position α- and β- from the carboxyl group) of the fatty acids’ chain. This outcome is consistent with the results of other studies [39] concerning the effect of UAE parameters on the fatty acid profile of the pumpkin lipid fraction, where less polar solvents and higher solvent volumes favor the recovery of lipid constituents, such as fatty acids and choline.

Following a similar pattern, the discrimination of Group 1 (in green) and Group 3 (in red) (Figure 10a) was attributed to the same metabolites (Figure 10b). Valine, isoleucine, and leucine did not significantly contribute to the discrimination in Group 3 (Figure 10b), but the distribution of the content values (especially in the case of valine) was small (Figure 10c, green bars) due to the use of ethanol (solvent with a higher polarity), as the only extraction solvent of these samples. 

Although lactic acid, present in unripe apricots and fruit byproducts [40], is a discriminant metabolite of Group 1 (in green) (Figure 10b), the distinct extraction solvent systems used for the extracts of this group (while the rest of the extraction conditions were kept constant) (Table S1) resulted in a higher distribution of the samples around the median value (Figure 10c). 

Furthermore, it is interesting to note that the role of myo-inositol and fatty acids was equally significant for this classification when compared to the previous OPLS-DA model (i.e., Group 1 vs. Group 2). As reported by other research groups [36,37], the recovery of lipids and fatty acids, either extracted by UAE or MAE, is more efficient at higher extraction times than the ones applied (15 min) in the studied extracts of Group 1 (green dots) (Tables S2 and S5).

Additionally, it is more than expected that fructose plays a key role in Group 3 (red dots), which mainly contains MAE extracts, since the more polar solvents (ethanol), higher temperatures, and higher solvent/material ratios applied upheld sugar extraction [41,42,43]. However, due to the different extraction conditions for each run (Tables S2b and S5), a higher distribution of group samples was observed (Figure 10c, green bar).

Ultimately, the trend along the second principal component (PC2) resulted in the discrimination of DOE-extracts with medium (Group 2 in blue) and high (Group 3 in red) carotenoid contents (Figure 11a). Although, as stated before, Group 1 (low carotenoid content) presented a higher content of amino acids, valine, leucine, and lysine still play quite an important role in the discrimination of Group 2 (blue dots) and Group 3 (red dots) (Figure 11b). According to the box-plots in Figure 11c, valine, leucine, and lysine in Group 2 (red color bars) presented a higher content than in Group 3 (green bars). Group 2, comprised of UAE extracts whose extraction solvent mixture includes a less polar solvent (chloroform), shows a higher affinity to non-polar amino acids than ethanol, which is the solvent of Group 3 samples (Tables S2a and S5). 

The higher distribution of values in amino acid box-plots (Figure 11c, red bars) concerning the extracts of Group 2 (blue dots), may be attributed to the great variation in the extraction time used in each of the samples, which ranged from 5 to 35 min, while the US power and solvent/material ratio were kept at high values (≥600 W and ≥30 mL g^−1^, respectively). 

Despite the contribution of amino acids in the discrimination of the two groups, the major metabolites playing a crucial role in their separation were apricots’ mono- and di-saccharides [41,44] (Figure 11b). Group 3 (red dots) primarily includes MAE extracts. Higher extraction temperatures of MAE (~60 °C) and solvent/material ratios facilitate the extraction of mono-saccharides, such as glucose, xylose, and fructose, and di-saccharides, such as sucrose [42] (Tables S2b and S5). Moreover, the high US powers of UAE extracts in Group 2 (in blue) promote mono-saccharides’ (glucose and xylose) than di-saccharide recovery (Tables S2a and S5) [43]. The symmetrical distribution and the tight grouping of Group 2 and Group 3 samples in the relative box-plots of sugars (Figure 11c) are more likely a result of keeping the values of the MAE temperature (~60 °C) and UAE’s US power (600–675 W) high, but quite constant, among the extracts (Tables S2 and S5).

To conclude, a final extract with a lower carotenoid content and branched-chain amino acids can be obtained by using non-polar solvents in UAE or MAE. A product containing choline, unsaturated fatty acids, sugar alcohols, and medium carotenoids content can be delivered at a high US power and solvent volumes. Finally, a sugar- and carotenoid-rich extract is provided when MAE at a high MW power, higher temperatures (compared to UAE), and higher solvent volumes is applied. 

In conclusion, the NMR-based screening of extracts, which are delivered by different extraction methods at distinct extraction conditions, should be considered as a quite useful basic research tool for (a) enabling standardization and (b) directing a future large-scale extraction procedure towards the acquisition of extracts containing different bioactive components with ‘tailor-made’ biological activities.

## 3. Materials and Methods 

### 3.1. Reagents and Standards

Beta-carotene and *trans*-β-apo-8′-carotenal were purchased from Sigma-Aldrich (St. Louis, MO, USA). *trans*-Lutein and *trans*-zeaxanthin were acquired from Extrasynthese (Genay, France). All solvents tested were of analytical grade. Acetone was purchased from ChemLab (Zedelgem, Belgium), while chloroform, methanol, ethanol, and *n*-hexane were obtained from Merck (Darmstadt, Germany). Scharlau (Barcelona, Spain). Fluka (Darmstadt, Germany) and Fischer Chemical (Pittsburgh, PA, USA) provided LC-MS grade methanol, acetonitrile, and methyl-tert-butyl ether (MTBE).

### 3.2. Plant Material and Sample Preparation

Apricot pulp was kindly provided by Danais S.A. Fruit Processing Industry & Export Company (www.danais-sa.com). Apricot fruits of the ‘Bebekos’ variety, which represents 70% of Greek production, were collected from the region of Argos, Peloponnese, Greece, during June 2017. Apricot byproducts, which were generated during the processing and compression of raw fruits, mainly included skin and also flesh with a particle size of over 0.5 mm.

Apricot pulp was freeze dried in a ModulyoD Freeze Dryer, equipped with a Thermo Savant ValuPump VLP200 (Thermo Electron Corporation, Thermo Fischer, Waltham, MA, USA). Freeze-drying was selected as the pulp drying method since it protects sensitive metabolites from degradation during long-term storage. This method removes sample moisture that may produce undesirable chemical reactions and promote microbial growth [45]. Lyophilization is a requisite step for carotenoid extraction, as the water content of byproducts hinders the recovery of non-hydrophilic compounds from plant substrates [15]. Dried material was homogenized and powdered in a laboratory mill (Type ZM1, Retsch GmbH, Haan, Germany). Dry material and all samples and extracts were kept in glass jars and vials at −20 °C.

### 3.3. Extraction Instrumentation and Processes

The ultrasound-assisted extraction (UAE) process was carried out by a Vibra-Cell VCX 750 (20 kHz, 750 W) ultrasonics processor (Sonics and Materials Inc., Newtown, USA), equipped with a piezoelectric converter and 13 mm diameter probe fabricated from titanium alloy Ti–6Al–4V. The microwave-assisted extraction (MAE) process was performed by a CEM Focused Microwave System, Model Discover (CEM Corporation, Matthews, NC, USA), in an open vessel or focused microwave (FMAE) mode with a reflux system placed above the open cell. A Centrifuge CL30 (Thermo Scientific, Waltham, MA, USA) was employed for the classical extraction. All extraction experiments were conducted according to our previous works [18] and their steps are described in the Supplementary Data.

### 3.4. Construction of DOE Models

A two-level full factorial design, 2^3^, and a symmetrical 16-run three-level Box–Behnken design (BBD) were selected for screening and optimization purposes, respectively. DOE extraction factors were the (a) extraction time, X_1_ (min); (b) US/MW power, X_2_ (W); and (c) solvent/material ratio, X_3_ (mL g^−1^). The impact of the above extraction factors on the carotenoid content, measured by LC-MS/MS, was evaluated through an assessment of the factors’ main effects and interactions [18]. The analysis of DOE models is unbiased when extraction variables take coded normalized dimensionless values (x_1_, x_2_, x_3_) instead of their real values (X_1_, X_2_, X_3_), which are expressed in different physical units. The two-level full factorial and BBD real and normalized values are shown in Table 4. Data and graphs were delivered using the Statistica package (Version 12, Stat Soft, Inc., Tulsa, OK, USA). Measurements’ confidence level was set at 95% (*p*-values ≤ 0.05).

### 3.5. Identification and Quantitation of Apricot Pulp Carotenoids by Liquid Chromatography-Photodiode Array-Tandem Mass Spectrometry (LC-PDA-MS/MS)

Liquid chromatography (LC) instrumentation was a combination of (a) a quaternary pump, (b) an autosampler with a tray oven set at 10 °C (Accela, Thermo Scientific, Waltham, MA, USA), (c) an Acclaim C30 reversed-phase column (3 μm particle size, 150 × 2.1 mm i.d) thermostatted at 20 °C, and (d) a guard column. The injection volume was set at 5 μL and the mobile phase flow rate was set at 350 μL/min. Mobile phase solvents were (A) acetonitrile (ACN), (B) methanol (MeOH), and (C) methyl-tert-butyl ether (MTBE). The eluting gradient program was the following: 0–5 min (30% A, 70% B), 5.1–13 min (22.9% A, 65.8% B, and 11.3% C), 13.1–14 min (5% A, 75% B, and 20% C), 14–14.1 min (30% A, 70% B), and 14.10–20 min (30% A, 70% B). MeOH-MTBE 50:50 *v*/*v* was the injection solvent.

No modifiers were added to the mobile phase as acetic and formic acid reduced m/z intensities and ammonium acetate was not preferred for lipid compounds [46]. The photodiode array (PDA) detector was set at 424, 445, and 455 nm. The ion trap mode of the LTQ Orbitrap Velos mass spectrometer (Thermo Scientific, USA) was used for mass spectrometry (MS) identification. MS/MS measurements were performed in a positive mode using an atmospheric pressure chemical ionization (APCI) source at a mass scan width of 150–650 *m*/*z*.

Source parameters were optimized by applying a Plackett–Burman design (Table S6, Figure S7), as described in the supplementary data. Polyester filters (15 mm diameter, 0.45 μm pore size, Macherey-Nagel, Duren, Germany) was utilized for sample filtration. LC-MS/MS data were processed with Xcalibur software (version 2.1, Thermo Scientific, Waltham, MA, USA). 

The development [47,48] and validation (Tables S7 and S8) of the LC-PDA-MS/MS [18,24,49,50,51] method are fully described in the supplementary data.

### 3.6. NMR Spectroscopy for the Elucidation of Non-Carotenoid Secondary Metabolites of Apricot Extracts

A Varian-600 MHz NMR spectrometer (Varian, Palo Alto, CA, USA) was used for acquiring NMR spectra. All spectra were obtained at an ambient temperature (25 °C) with a triple resonance {HCN} probe.

#### 3.6.1. Sample Preparation and NMR Measurements

Twenty milligrams (20 mg) of apricot extract dry residue was dissolved in 550 μL of *d*_4_-methanol, and 50 μL of TSP 5 mM (internal standard) was then added. Samples were transferred to 5 mm NMR tubes.

A one dimension-Nuclear Overhauser effect spectroscopy (1D-NOE) pulse sequence was applied for ^1^H-NMR spectra. Spectra acquisition was performed at 128 transients collected with 128 K data points, a spectral width of 7163.9 Hz, a relaxation delay of 1 s, an acquisition time of 4.454 s, and a mixing time of 200 ms. The receiver gain was constant during all acquisitions.

Two dimensional (2D) experiments of gCOSY, gHMBCad, and gHSQCad, recorded at 25 °C, enabled the identification of extract metabolites. An analysis of 2D spectra was carried out using MestReNova v.14.1 software (Mestrelab Research, S.L., Santiago de Compostela, Spain). Metabolite elucidation was facilitated by 2D NMR spectra plus reported data and cross-referenced with the web-server metabolite database Metaboneer, which is an in-house fully automated metabolite identification platform [52].

#### 3.6.2. Data Reduction and Spectral Alignment

All spectra were processed by MestReNova v.14.1 software for phasing, baseline correction, removal of the methanol peak, binning into spectral buckets of 0.001 ppm, and normalization to the reference compound standardized area. All spectra were converted to ASCII format and then imported into MATLAB (R2006a, Mathworks, Inc. 2006, Natick, MA, USA), where they were aligned using the Correlation Optimized Warping (COW) method.

### 3.7. Multivariate Data Analysis

The SIMCA-P version 14.0 (Umetrics, Umeå, Sweden) was used for the statistical processing of NMR data from apricot pulp analysis. The first step was the acquisition of a general overview and the visualization of trends and outliers among apricot extracts by applying the exploratory PCA analysis.

Further analysis of the NMR dataset occurred with supervised OPLS-DA models, in order to estimate the between-class and within-class variation. All models were derived at a 95% confidence level after being mean-centered with Pareto scaling, which only includes low/medium intensity metabolites in the model if they display systematic variation.

The extraction variables and conditions that were singled out for their class discriminating power were revealed from loading plots. Models’ goodness-of-fit and predictive ability were evaluated by R^2^ (0 ≤ R^2^ ≤ 1) and Q^2^ (0 ≤ Q^2^ ≤ 1) values, respectively. R^2^ refers to the data variance interpreted by the model, while cumulative Q^2^ describes the variance of the data which are predictable by the model. In OPLS-DA models, the statistical importance of R^2^ and Q^2^ is evaluated through response permutation testing (999 permutations employed in our study) and a receiver operating characteristic (ROC) curve. In a permutation test plot, a model is valid when the intercept of the Q^2^ regression line is lower than zero and the intercept of the R^2^ regression line is crucially lower than that of the original [53]. A model is also considered significant when ROC values are ≥0.75. S-line plots highlighted the metabolites that contributed to DOE extract discrimination.

## 4. Conclusions

In recent years, coping with the problem of agro-byproduct accumulation and disposal has created new potential for the profitable and eco-friendly management of food and agro-waste through the use of innovative analytical strategies. In the current project, the results of the implementation of UAE and MAE for the recovery of carotenoids and other bioactive co-extractives from apricot pulp are summarized below.

Carotenoids from apricot byproducts were obtained in higher amounts when MAE was applied. Compared to classic extraction (the Folch method), high energy extractions provided higher (19-times more in the case of MAE) carotenoid yields. 

Reflecting on the concept of transforming a byproduct to high-added value products, the potential of NMR spectroscopy for apricot byproducts was explored. 

Despite the fact that MAE provided higher carotenoid yields, the main co-extractives of these samples were sugars, whose removal and clean-up could be more challenging and laborious than for other co-extractives.

Furthermore, extracts of Group 2 (Table S5), mostly obtained by UAE at a high US power and solvent/material ratio, can result in an extract with combined biological activities due to their significant content of carotenoids (improving eye health, immune-modulating properties, anti-allergic, anti-aging, and sun-protective activity) [13,54], choline (enhancing brain health and cognitive function, affecting detoxification pathways and organs, such as the liver and kidneys) [55,56,57], myo-inositol (promoting female fertility, treating polycystic ovary syndrome, reducing anxiety, and restoring insulin resistance) [58,59,60], and unsaturated fatty acids (health-promoting agents against fat burning, affecting inflammation, steroid signaling, and membrane-bound protein functions, participating in glucose/lipid metabolism) [61,62,63].

Although MAE provided extracts with higher carotenoids yields, in the case of a prospective scaling-up of high energy extraction processes in the field of nutraceuticals or cosmetics, UAE will probably emerge as a more suitable approach, as it delivered extracts with a significant carotenoid content, rich in other bioactive constituents (choline, myo-inositol, and fatty acids), and with a lower sugar concentration, compared to MAE extracts. 

Therefore, summarizing the results of this research, high energy extraction methods emerge as an attractive alternative for tackling any challenges or drawbacks of the current large-scale extraction methods from an industrial point of view, as they provide high quality extracts. These results should be considered as an elementary index or starting point for the development of nutraceutical supplements. 

This study is the first to integrate high energy extraction techniques, DOE models, LC-MS/MS, and NMR spectroscopy, in order to correlate extracts of different carotenoid yields with particular secondary metabolites by adjusting UAE or MAE parameters and to allow imminent standardization of the extraction procedure to obtain multi-targeted and multi-functional natural extracts. In that way, high energy extractions can be re-evaluated and upgraded to reliable sample preparation steps in the field of plant metabolomics.

## Figures and Tables

**Table 1:** Optimal values of UAE and MAE parameters for carotenoid recovery from apricot byproducts.

Extraction Parameters	Optimal Values	
	UAE	MAE
Extraction solvent (*v*/*v*)	Methanol:chloroform 1:1	Ethanol
Extraction time (min)	10	20
US/MW power (W)	600	120
Solvent/material ratio (mL g^−1^)	35	45
US pulse sequence (s)/MW ramping time (min)	15 ON 5 OFF	0
Extraction yield (mg of carotenoids 100 g^−1^ dry sample) (±stdev), n = 3 ^1^	11.12 (±0.34)	19.28 (±0.27)

^1^ n: number of sample replicates measured under repeatability conditions.

**Table 2:** Carotenoid content of apricot pulp determined by LC-MS/MS.

Carotenoids Content (mg 100 g^−1^ Dry Sample), v = 3 ^1^	Optimized UAE Extract	Optimized MAE Extract	Folch Extract
β-Carotene	7.72 (±0.98)	19.7 (±1.6)	1.44 (±0.87)
Zeaxanthin	0.71 (±0.33)	0.66 (±0.25)	0.020 (±0.041)
Lutein	0.82 (±0.19)	0.82 (±0.12)	0.07 (±0.24)
Final carotenoid content expressed in mg kg^−1^ of raw apricot pulp sample (N = 3) ^2^			
Average weight (g) of raw apricot pulp samples (±stdev), n = 10 ^3^	17.1 (±2.1)		
Average weight (g) of lyophilized apricot pulp samples (±stdev), n = 10 ^3^	3.23 (±0.39)		
Average moisture (%) of raw apricot pulp, n = 10 ^3^	81.25		
	UAE Extracts	MAE Extracts	Folch Extract
β-Carotene content (mg kg^−1^ raw apricot pulp) (±stdev)	14.58 (±0.98)	37.2 (±1.6)	2.72 (±0.87)
Zeaxanthin content (mg kg^−1^ raw apricot pulp) (±stdev)	1.34 (±0.33)	1.25 (±0.25)	0.038 (±0.041)
Lutein content (mg kg^−1^ raw apricot pulp) (±stdev)	1.55 (±0.19)	1.55 (±0.12)	0.13 (±0.24)

^1^ Number of LC-MS/MS replicates, ^2^ number of extraction replicates, and ^3^ number of samples.

**Table 3:** Characteristic ^1^H-NMR peaks of apricot byproduct extracts identified in the principal component analysis (PCA) groups.

Compounds	^1^H Chemical Shift	Peak Multiplicity ^1^
Valine	0.99, 1.04, 2.28	(d), (d), (m)
Leucine	0.98, 0.96	(d, *J* = 7.5), (d, *J* = 7.5)
Isoleucine	0.94, 1.01, 1.25, 1.45, 1.96, 3.66	(t), (d), (m), (m), (m), (m)
Alanine	1.48	(d)
Lysine	1.61	(t)
Choline	3.10	(s)
Fatty acids	1.26	(m)
Myo-inositol	3.67, 3.78	(t), (t)
Malic acid	2.68, 2.78	(dd), (dd)
Lactic acid	1.34	(d)
Formic acid	8.40	(s)
Fructose	3.53, 4.04	(t), (t)
Sucrose	4.18, 5.39	(d), (d, *J* = 3.9)
Glucose	5.12	(d)
Xylose	5.07	(d)

^1^ (s): single peak, (d): doublet, (dd): doublet of doublets, (t): triplet, (m): multiplet, and *J*: coupling constant.

**Table 4:** Normalized and real values of UAE/MAE experimental factors for a two-level full factorial design (2^3^) and Box–Behnken design (BBD) for apricot byproducts.

Coded Values	−1	0	+1
	2^3^ design		
	UAE		
Extraction time (X_1_, min)	5		35
US power (X_2_, W)	375		675
Solvent/material ratio (X_3_, mL g^−1^)	10		35
	MAE		
Extraction time (X_1_, min)	5	-	30
MW power (X_2_, W)	70	-	200
Solvent/material ratio (X_3_, mL g^−1^)	20	-	60
	BBD		
	UAE		
Extraction time (X_1_, min)	10	20	30
US power (X_2_, W)	577	622	675
Solvent/material ratio (X_3_, mL g^−1^)	25	30	35
	MAE		
Extraction time (X_1_, min)	5	10	20
MW power (X_2_, W)	60	90	130
Solvent/material ratio (X_3_, mL g^−1^)	45	65	55

## Supplementary Materials

The following are available online at https://www.mdpi.com/1420-3049/25/11/2702/s1, Figure S1: Contour plots of (**a**) UAE extraction time vs. US power; (b) UAE US power vs. solvent/material ratio; (c) UAE solvent/material ratio vs. extraction time; (d) MAE extraction time vs. MW power; (e) MAE MW power vs. solvent/material ratio; and (f) MAE solvent/material ratio vs. extraction time; Figure S2: Pareto charts of (a) UAE and (b) MAE, where 1 = extraction time, 2 = US or MW power, 3 = solvent/material ratio, L = linear terms, and Q = quadratic terms; Figure S3: 1D-NMR spectrum of a characteristic apricot extract (i.e., UAE ethanol-acetone extract): (a) ^1^H-NOESY spectrum; (b) chemical shifts region of amino acids, lactic acid, and fatty acids; (c) chemical shifts region of myo-inositol, choline, and malic acid; (d) chemical shifts region of sugars; Figure S4: 2D spectra of a characteristic extract of (a) Group 1 gCOSY; (b) Group 1 gHSQCad; (c) Group 2 gCOSY; (d) Group 2 gHSQCad; (e) Group 3 gCOSY; and (f) Group 3 gHSQCad; Figure S5: Contribution plot of Folch samples; Figure S6: Permutation testing and ROC curves of OPLS-DA models: (a) Group 1 vs. 2+3; (b) Group 1 vs. 2; (c) Group 1 vs. 3; and (d) Group 2 vs. 3; Figure S7: Plackett–Burman design: Normal probability plot of the effect of APCI parameters on the β-carotene intensity; Table S1: UAE and MAE extraction yields of different solvent systems; Table S2: Randomized experimental runs and carotenoid extraction yield of 23 and BBD models of (a) UAE and (b) MAE; Table S3: ANOVA table of (a) 2^3^ design and (b) BBD model for UAE and MAE of apricot pulp carotenoids; Table S4: Predicted and observed extraction yields of apricot pulp at optimal experimental combinations proposed by the BBD model; Table S5: Apricot pulp sample classification produced by PCA models; Table S6: Plackett–Burman design: Coded and real values of APCI parameters; Table S7. Analytical figures of merit of LC-MS/MS for apricot pulp carotenoid determination; Table S8. Precision, accuracy, and process recovery of the LC-MS/MS method for apricot pulp carotenoid determination.
