# Supplementary material for: Expanding the Role of Sub-Exploited DOE-High Energy Extraction and Metabolomic Profiling towards Agro-Byproduct Valorization: The Case of Carotenoid-Rich Apricot Pulp
Source: Molecules. 2020 Jun 11;25(11):2702. doi: 10.3390/molecules25112702 (PMC7321327; doi:10.3390/molecules25112702)
Supplement: Supplementary file 1 [file molecules-25-02702-s001.pdf]

molecules

MDPI

Supplementary Materials for

Expanding the Role of Sub-Exploited DOE-High Energy Extraction and Metabolomic Profiling towards Agro-Byproduct Valorization: The Case of Carotenoid-Rich Apricot Pulp

Table S1. UAE and MAE extraction yields of different solvent systems.

Extraction Solvent (v/v)	Extraction Yield (mg of carotenoids 100 g^{-1} dry sample) (±stdev, n = 3) [1]	
	UAE [2]	MAE [3]
**Chloroform-Methanol 1:1**	9.39(±0.15)	6.97(±0.27)
**Chloroform-Methanol 2:1.**	0.94(±0.092)	3.33(±0.35)
**Chloroform**	0.34(±0.10)	1.192(±0.095)
**Ethanol-Acetone 1:1**	6.13(±0.23)	10.61(±0.86)
**Ethanol**	5.03(±0.31)	11.09(±0.98)
**Acetone**	0.75(±0.17)	0.48(±0.26)
***n*-Hexane-Acetone 1:1**	2.66(±0.20)	0.84(±0.31)
***n*-Hexane-Acetone-Ethanol 2:1:1**	2.02(±0.067)	0.27(±0.15)

[1] n, the number of replicates; [2] UAE conditions: 15 min, 487.5 W, 30 °C, 20 mL g^{-1}, 15 s ON/5 s OFF;.[3] MAE conditions: 15 min, 150 W, temperature set at the boiling point of each solvent, 20 mL g^{-1}, ramping time set at zero.

Table S2. Randomized experimental runs and carotenoid extraction yield of 2^3 and BBD models of: **(a)** UAE; **(b)** MAE.

(a)

Standard Run	Coded Combinations (x_1, x_2, x_3)	Extraction Yield for UAE (mg of carotenoids 100 g^{-1} dry sample)
	2^3 design	
**2**	-1,-1,+1	5.65
**3**	-1,+1,-1	8.53
**6**	+1,-1,+1	7.84
**4**	-1,+1,+1	15.91
**5**	+1,-1,-1	4.50
**8**	+1,+1,+1	29.18
**1**	-1,-1,-1	6.64
**7**	+1,+1,-1	3.21
	BBD model	
**16**	0,0,0	11.35
**4**	-1,+1,0	5.74
**12**	0,+1,+1	8.65
**3**	-1,+1,0	10.45
**13**	0,0,0	15.77
**6**	+1,0,-1	8.22
**11**	0,-1,+1	3.82
**8**	+1,0,+1	6.46
**14**	0,0,0	11.29
**1**	-1,-1,0	11.56
**10**	0,+1,-1	9.79
**15**	0,0,0	11.48
**7**	-1,0,+1	12.15
**9**	0,-1,-1	13.05
**5**	-1,0,-1	6.86
**2**	+1,-1,0	7.70

(b)

Standard Run	Coded Combinations (x_1, x_2, x_3)	Extraction Yield for MAE (mg of carotenoids 100 g^{-1} dry sample)
	2^3 design	
**2**	-1,-1,+1	23.80
**3**	-1,+1,-1	16.05
**6**	+1,-1,+1	19.19
**4**	-1,+1,+1	14.00
**5**	+1,-1,-1	13.01
**8**	+1,+1,+1	11.76
**1**	-1,-1,-1	12.14
**7**	+1,+1,-1	16.29
	BBD model	
**16**	0,0,0	18.37
**4**	-1,+1,0	18.91
**12**	0,+1,+1	15.00
**3**	-1,+1,0	17.81
**13**	0,0,0	13.26
**6**	+1,0,-1	20.27
**11**	0,-1,+1	18.06
**8**	+1,0,+1	18.05
**14**	0,0,0	14.40
**1**	-1,-1,0	14.74
**10**	0,+1,-1	19.35
**15**	0,0,0	18.16
**7**	-1,0,+1	18.11
**9**	0,-1,-1	7.52
**5**	-1,0,-1	12.03
**2**	+1,-1,0	10.44

Table S3. ANOVA table of: **(a)** 2^3 design; **(b)** BBD model for UAE and MAE of apricot pulp carotenoids.

(a)

**Factors**		**Sum of Squares (SS)**		***F*-value**		***p*-value**	
**UAE**	**MAE**	**UAE**	**MAE**	**UAE**	**MAE**	**UAE**	**MAE**
x_2	x_1	*129.69*	4.13	*9.45*	5.45	*0.054*	0.15
x_3	x_2	*159.38*	12.57	*11.61*	16.59	*0.042* [1]	0.055
x_1x_3	x_3	*65.70*	15.83	*4.79*	20.89	*0.12*	0.045 [1]
x_2x_3	x_1x_3	*120.13*	7.93	*8.75*	10.47	*0.060*	0.084
	x_2x_3		74.54		98.36		0.010 [1]
		**UAE**	**MAE**				
**Pure Error (Degrees of Freedom)**		*41.17 (3)*	1.52 (3)				
**Total SS (Degrees of Freedom)**		*516.09 (7)*	116.52 (7)				

[1] Factors with *p*-value ≤ 0.05

(b)

Factors		Sum of Squares (SS)		*F*-value		*p*-value	
**UAE**	**MAE**	**UAE**	**MAE**	**UAE**	**MAE**	**UAE**	**MAE**
x_1^2	x_1^2	*16.10*	1.63	*5.93*	0.54	*0.051*	0.48
x_1	x_2	*23.06*	58.20	*8.49*	19.18	*0.027* [1]	0.0024 [1]
x_2^2	x_2^2	*10.24*	18.83	*3.77*	6.20	*0.10*	0.037
x_3^2	x_1x_2	*16.69*	7.97	*6.14*	2.63	*0.048* [1]	0.14
$x_1x_2^2$	$x_1x_2^2$	*2.23*	22.20	*0.82*	7.32	*0.43*	0.027 [1]
$x_1^2x_2$	x_1x_3	*1.94*	24.16	*0.71*	7.96	*0.40*	0.022 [1]
x_1x_3	x_2x_3	*12.41*	58.43	*4.57*	19.25	*0.076*	0.0023 [1]
$x_1^2x_3$		*29.41*		*10.83*		*0.017* [1]	
x_2x_3		*16.37*		*6.03*		*0.04* [1]	
		**UAE**	**MAE**	**UAE**	**MAE**	**UAE**	**MAE**
**Lack of Fit (Degrees of Freedom)**		*1.77* *(3)*	3.95 (5)	0.12 (3)	0.12 (5)	*0.941* *(3)*	0.979 (5)
**Pure Error (Degrees of Freedom)**		*14.52* *(3)*	20.32 (3)				
**Total SS (Degrees of Freedom)**		*142.52* *(15)*	195.45 (15)				

[1] Factors with *p*-value ≤ 0.05

Table S4. Predicted and observed extraction yields of apricot pulp at optimal experimental combinations proposed by BBD model.

UAE	Extraction Time (min)	US Power (W)	Solvent/Material Ratio (mL g^{-1})	Predicted Extraction Yield (mg of carotenoids 100 g^{-1} dry sample)	Experimental Extraction Yield (mg of carotenoids 100 g^{-1} dry sample) (±stdev)
**Run A**	15	600	25	12.43	8.46(±0.21)
**Run B**	10	600	35	10.83	11.12(±0.34) [1]
**Run C**	20	600	25	13.75	9.38(±0.15)
**MAE**	**Extraction Time (min)**	**MW Power (W)**	**Solvent/Material Ratio (mL g^{-1})**	**Predicted Extraction Yield (mg of carotenoids 100 g^{-1} dry sample)**	**Experimental Extraction Yield (mg of carotenoids 100 g^{-1} dry sample) (±stdev)**
**Run A**	20	120	45	19.40	19.28(±0.27) [1]
**Run B**	20	120	60	19.51	17.22(±0.38)
**Run C**	20	140	60	18.98	15.29(±0.26)

[1] Optimal UAE and MAE experimental combination.

Table S5. Apricot pulp samples classification produced by PCA models.

Low Extraction Yield (≤5 mg carotenoids 100 g^{-1} dry sample)	Medium Extraction Yield (5–15 mg carotenoids 100 g^{-1} dry sample)	High Extraction Yield (≥15 mg carotenoids 100 g^{-1} dry sample)
MAE_$CHCl_3$-MeOH_2:1	MAE_$CHCl_3$-MeOH_1:1	MAE_Run 2_2^3 design
MAE_$CHCl_3$	MAE_EtOH-Acetone	MAE_Run 3_2^3 design
MAE_Acetone	MAE_EtOH	MAE_Run 8_2^3 design
MAE_*n*-Hexane-Acetone	UAE_EtOH-Acetone	MAE_Run 6_BBD
MAE_*n*-Hexane-Acetone-EtOH 2:1:1	UAE_EtOH	MAE_Run 10_BBD
UAE_$CHCl_3$-MeOH_2:1	UAE_$CHCl_3$-MeOH_1:1	MAE_Run 12_BBD
UAE_$CHCl_3$	UAE_Run 3_2^3 design	UAE_Run 4_2^3 design
UAE_Acetone	UAE_Run 6_2^3 design	UAE_Run 8_2^3 design
UAE_*n*-Hexane-Acetone	UAE_Run 14_BBD	MAE_Optimal values
UAE_*n*-Hexane-Acetone-EtOH 2:1:1	UAE_Optimal values	
UAE_Run 11_BBD		
Folch		

Table S6. Plackett-Burman design: Coded and real values of APCI parameters.

MS factors		*Coded values*	
	-1	0 [1]	1
		Real values	
**S-LENS RF Amplitude (V)**	55	63	70
**Vaporizer Temperature (°C)**	300	375	450
**Sheath Gas Flow Rate (a.u.)**	25	38	50
**Auxiliary Gas Flow Rate (a.u.)**	10	8	5
**Sweep Gas Flow Rate (a.u.)**	10	8	5
**Discharge Current (μA)**	9	7	5
**Capillary Temperature (°C)**	150	225	300

[1] Center points added to two-level Plackett-Burman design to detect curvature in the response and to evaluate variability by avoiding repetitions at the corner points.

Table S7. Analytical figures of merit of LC-MS/MS for apricots pulp carotenoids determination.

Analytical Figures of Merit	UAE			MAE		
	**β-Carotene**	**Zeaxanthin**	**Lutein**	**β-Carotene**	**Zeaxanthin**	**Lutein**
**Concentration Range (μg mL^{-1})**	0.5-15	5-15	0.5-15	2.5-20	0.5-15	0.5-15
**Slope (a) (±s_a)**	0.84 (±0.13)	0.367 (±0.070)	0.59 (±0.10)	0.633 (±0.093)	1.1 (±0.17)	0.751 (±0.049)
**Intercept (b) (±s_b)**	1.0 (±1.1)	-0.75 (±0.74)	-0.47 (±0.84)	0.1 (±1.1)	-0.2 (±1.5)	-0.54 (±0.41)
**LOD (μg mL^{-1})**	0.75	1.59	0.19	0.85	0.40	0.25
**LOQ (μg mL^{-1})**	2.28	5.31	0.63	2.58	1.21	0.75
**LOD/LOQ Concentration Range (μg mL^{-1})**	0.25-1.25	1.0-7.5	0.25-1.25	0.75-2.7	0.25-1.25	0.5-1.5
**R^2**	0.931	0.901	0.920	0.939	0.928	0.987
**Matrix Effect (ME) (%)**	219.32	198.92	77.44	165.27	253.20	230.00

Table S8. Precision, accuracy and process recovery of LC-MS/MS method for apricot pulp carotenoids determination.

**Carotenoids**	**UAE QC levels**		
β-Carotene	**1.0 μg mL^{-1} (n = 3) [2]**	**5.0 μg mL^{-1} (n = 3) [2]**	**12.5 μg mL^{-1} (n = 3) [2]**
**Intra-Day Precision (RSD$_r$%)**	10.82	10.42	7.21
**Inter-Day Precision (RSD$_r$%), N = 3 [1]**	15.05	9.80	6.69
**Accuracy**	83.00	106.00	94.53
**Process Recovery at Spike Level 5.0 μg mL^{-1}**		119.56	
Zeaxanthin	**7.5 μg mL^{-1} (n = 3) [2]**	**10.0 μg mL^{-1} (n = 3) [2]**	**12.5 μg mL^{-1} (n = 3) [2]**
**Intra-Day Precision (RSD$_r$%)**	12.80	8.66	7.34
**Inter-Day Precision (RSD$_r$%), N = 3 [1]**	12.01	7.77	6.53
**Accuracy**	109.80	79.2	104.60
**Process Recovery at Spike Level 10.0 μg mL^{-1}**		67.92	
Lutein	**1.0 μg mL^{-1} (n = 3) [2]**	**5.0 μg mL^{-1} (n = 3) [2]**	**12.5 μg mL^{-1} (n = 3) [2]**
**Intra-Day Precision (RSD$_r$%)**	10.81	9.96	6.54
**Inter-Day Precision (RSD$_r$%), N = 3**	14.69	7.10	5.18
**Accuracy**	104.00	96.40	109.73
**Process Recovery at Spike Level 5.0 μg mL^{-1}**		38.51	
**Carotenoids**	**MAE QC levels**		
β-Carotene	**5.0 μg mL^{-1} (n = 3) [2]**	**10.0 μg mL^{-1} (n = 3) [2]**	**15.0 μg mL^{-1} (n = 3) [2]**
**Intra-Day Precision (RSD$_r$%)**	8.45	4.86	7.31
**Inter-Day Precision (RSD$_r$%), N = 3 [1]**	9.19	4.25	5.79
**Accuracy**	86.80	89.80	86.70
**Process Recovery at Spike Level 5.0 μg mL^{-1}**		97.91	
Zeaxanthin	**1.0 μg mL^{-1} (n = 3) [2]**	**5.0 μg mL^{-1} (n = 3) [2]**	**12.5 μg mL^{-1} (n = 3) [2]**
**Intra-day precision (RSD$_r$%)**	14.14	2.23	4.39
**Inter-day precision (RSD$_r$%), N = 3 [1]**	11.67	8.91	10.95
**Accuracy**	100.80	87.80	86.60
**Process recovery at spike level 5.0 μg mL^{-1}**		93.77	
Lutein	**1.0 μg mL^{-1} (n = 3) [2]**	**5.0 μg mL^{-1} (n = 3) [2]**	**12.5 μg mL^{-1} (n = 3) [2]**
**Intra-Day Precision (RSD$_r$%)**	9.92	9.78	4.91
**Inter-Day Precision (RSD$_r$%), N = 3 [1]**	11.61	9.92	11.98
**Accuracy**	106.60	108.20	101.40
**Process Recovery at Spike Level 5.0 μg mL^{-1}**		116.71	

[1] N: the number of consecutive days required for inter-day precision determination; [2] n: the number of QC replicates.

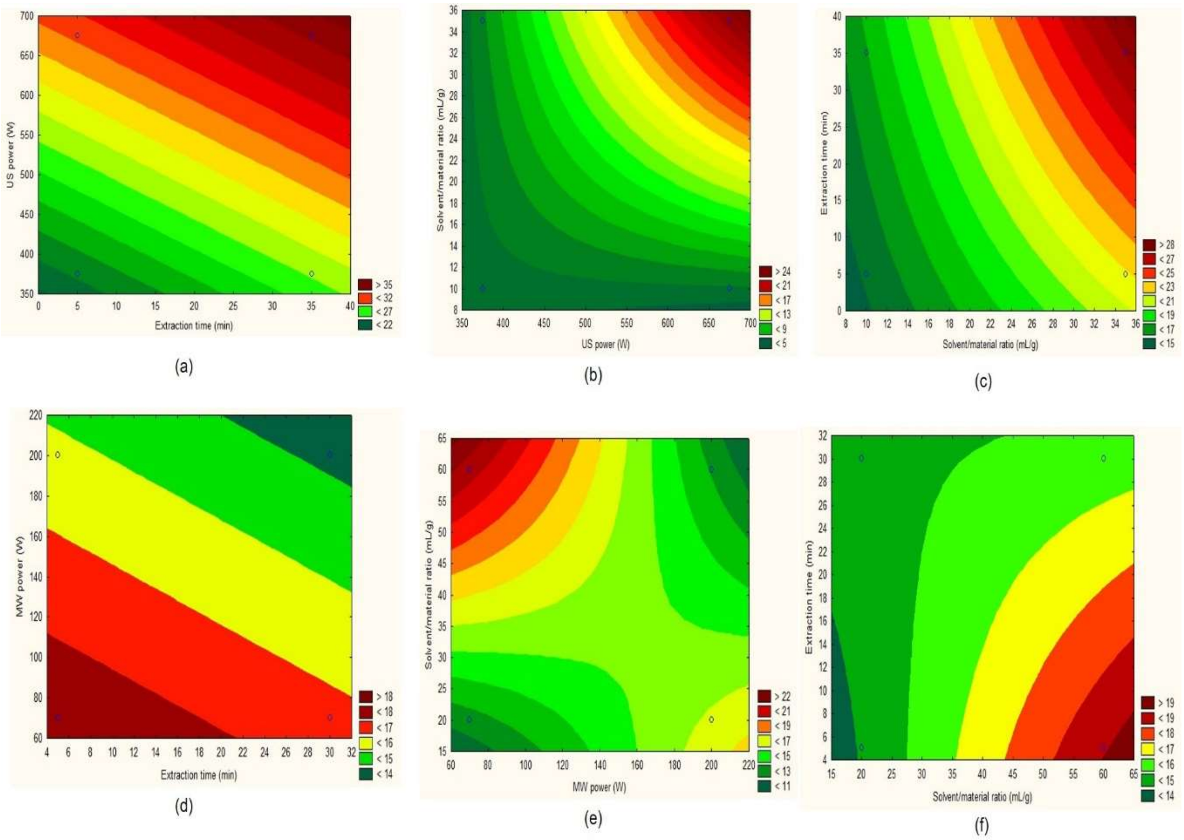

Figure S1. Contour plots of: (**a**) UAE extraction time vs US power; (**b**) UAE US power vs solvent/material ratio; (**c**) UAE solvent/material ratio vs extraction time; (**d**) MAE extraction time vs MW power; (**e**) MAE MW power vs solvent/material ratio; (**f**) MAE solvent/material ratio vs extraction time.

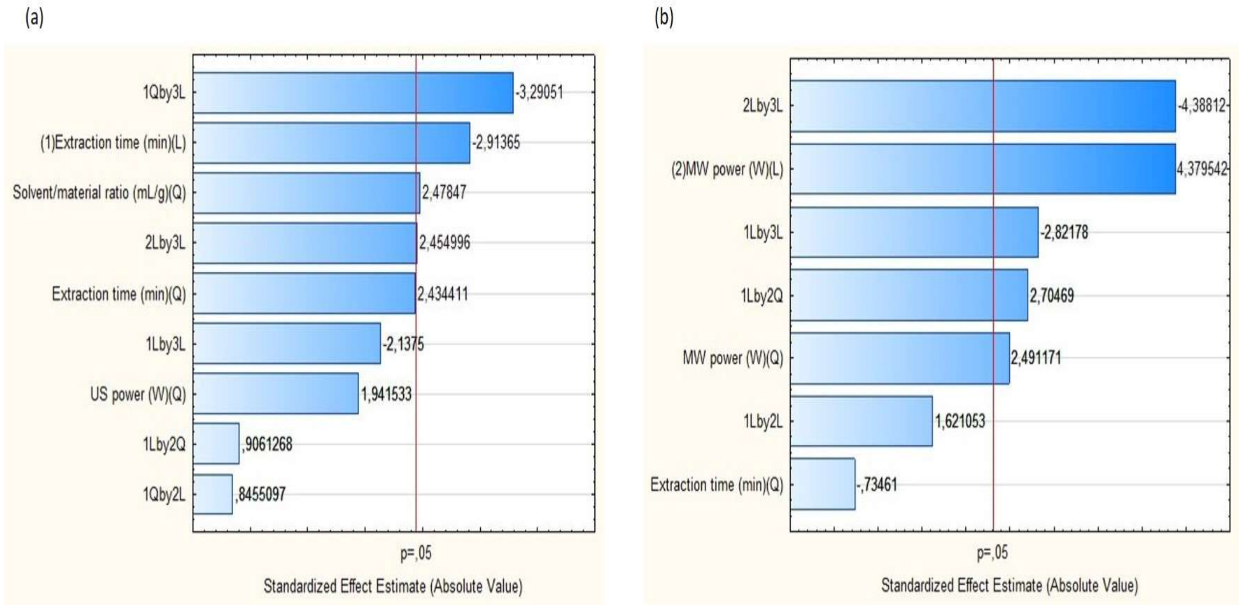

Figure S2. Pareto charts of: (**a**) UAE; (**b**) MAE, where 1 = extraction time, 2 = US or MW power, 3 = Solvent/material ratio, L = linear terms, Q = quadratic terms.

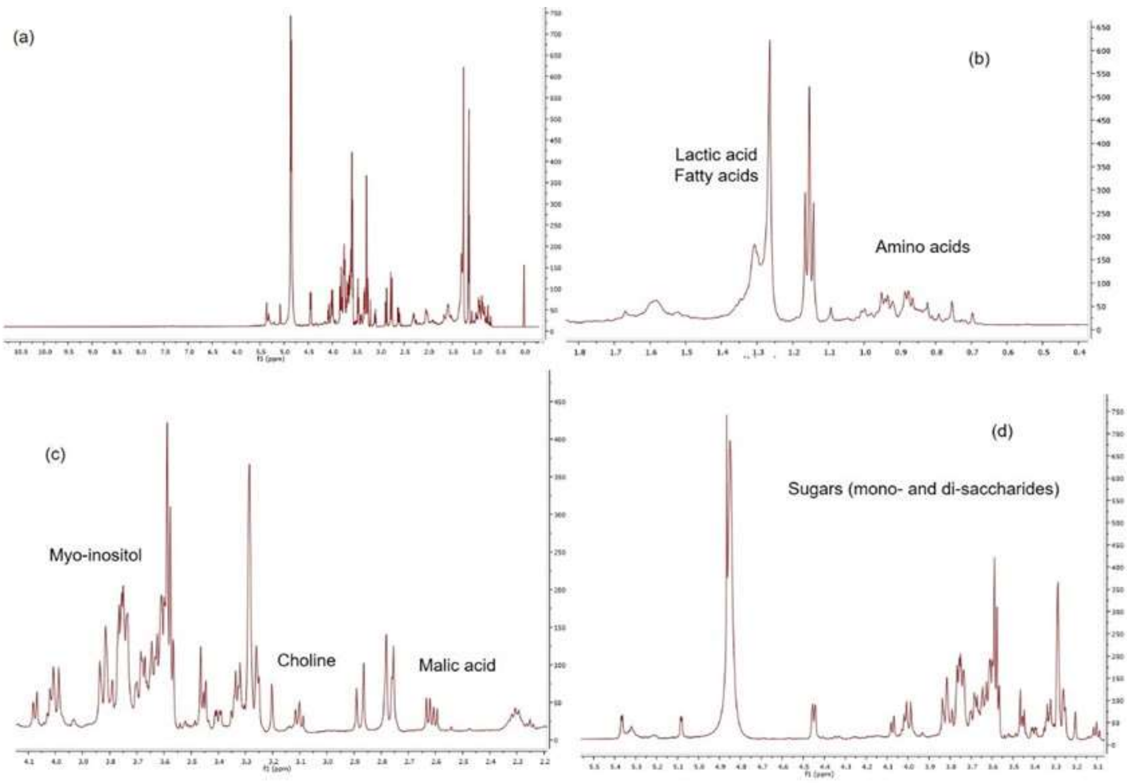

Figure S3. 1D NMR spectrum of a characteristic apricot extract (i.e. UAE Ethanol-Acetone extract): (**a**) 1H NOESY spectrum; (**b**) Chemical shifts region of amino acids, lactic acid and fatty acids; (**c**) Chemical shifts region of myo-inositol, choline and malic acid; (**d**) Chemical shifts region of sugars.

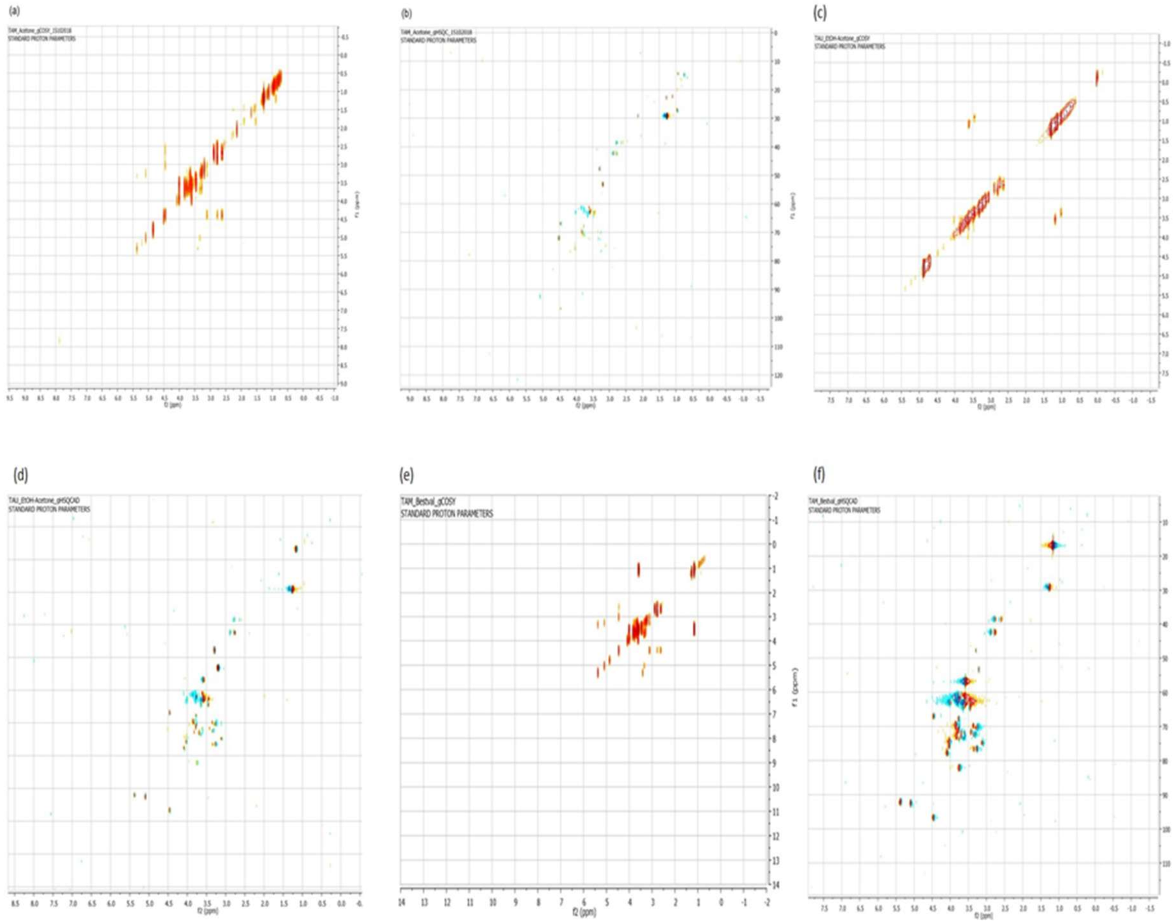

Figure S4. 2D spectra of a characteristic extract of: (**a**) Group 1 gCOSY; (**b**) Group 1 gHSQCad; (**c**) Group 2 gCOSY; (**d**) Group 2 gHSQCad; (**e**) Group 3 gCOSY; (**f**) Group 3 gHSQCad.

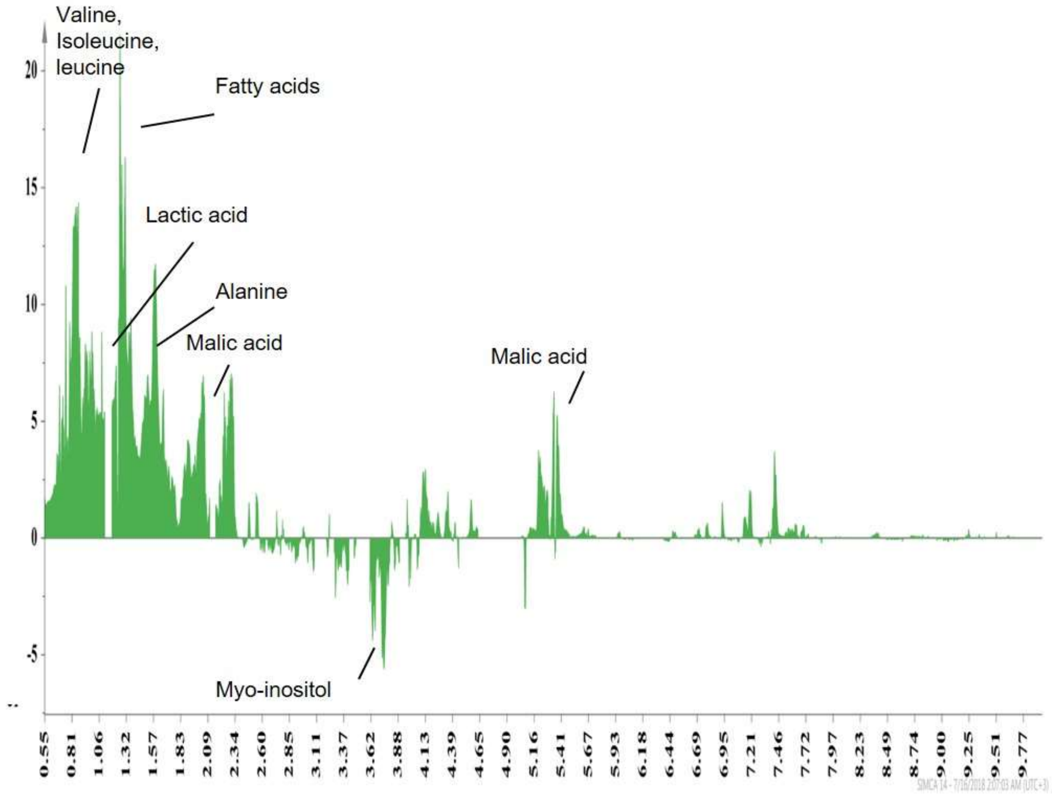

Figure S5. Contribution plot of Folch samples.

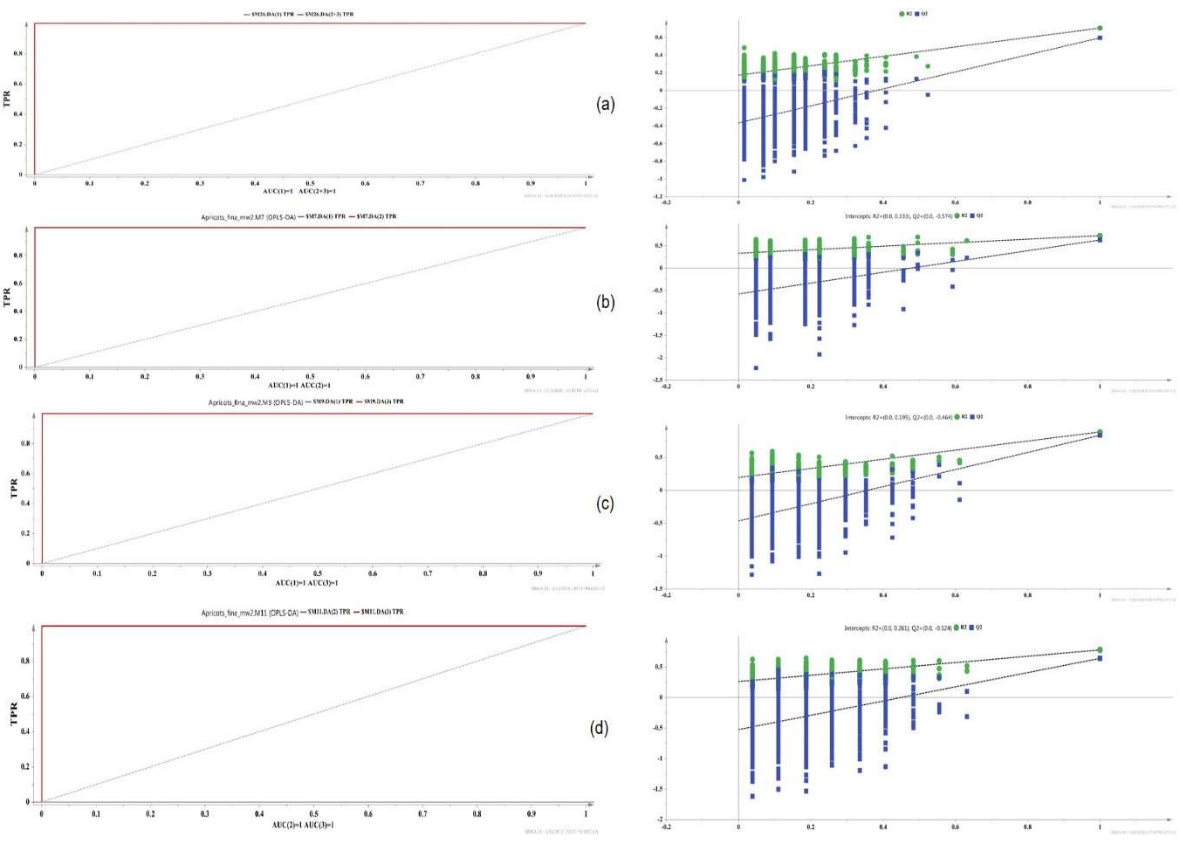

Figure S6. Permutation testing and ROC curves of OPLS-DA models: (**a**) Group 1 vs 2+3 (**b**) Group 1 vs 2 (**c**) Group 1 vs 3 (**d**) Group 2 vs 3.

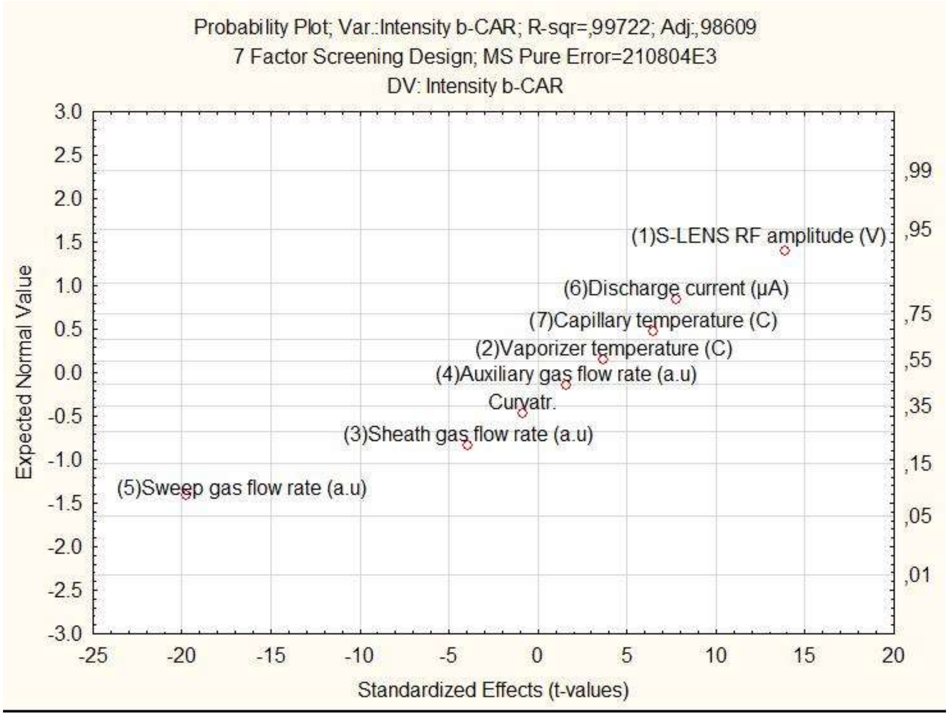

Figure S7. Plackett-Burman design: Normal probability plot of the effect of APCI parameters on β-carotene intensity.

Analytical Methods Description

1.1. Extraction Procedures

Various extraction solvents were applied for carotenoids UAE and MAE. Three grams (3 g), for UAE, and one gram (1 g), for MAE, of apricot powder was used. Methanol, ethanol, chloroform, n-hexane, acetone and their mixtures were the extraction solvents tested.

The applied classical extraction was a modified 3-step Folch method with a 12:1 (v/w) solvent/material ratio described in our previous works [18].

Extraction samples were centrifuged at 8000 rpm for 20 min. A rotary evaporator at 50 °C was used for acquiring dry residues of extracts. The obtained lipid fraction was diluted in 8 mL of extraction solvent. Next, a volume aliquot until 20 mg of carotenoid extract was flushed with N_2 stream. Lastly, N_2 dry residues was dissolved in certain final volumes of methanol:MTBE 1:1 *v*/*v* mixture (for LC-PDA-MS/MS) or deuterated solvents (for NMR). All experiments were performed in triplicate.

1.2. APCI Source Parameters Optimization

Screening of the seven APCI parameters revealed that carotenoids' intensity was higher at high levels of S-LENS RF amplitude, vaporizer and capillary temperature and low levels of sheath, auxiliary and sweep gas flow. Normal probability plots (Supplementary data, Figure S1b) identified S-LENS RF amplitude and sweep gas flow rate as the parameters affecting most carotenoids intensity. A further optimization led to the final optimal values, which were determined as: S-LENS RF amplitude level = 63%, vaporizer temperature = 400 °C, sheath gas flow rate = 25 a.u, auxiliary gas flow rate = 5 a.u, sweep gas flow rate = 0 a.u, discharge current = 4 μA and capillary temperature = 300 °C. ISO mass width was equal to 2.0. Collision energies for internal standard, β-carotene and zeaxanthin were set at 40 eV, while for lutein was adjusted at 35 eV.

1.3. Development and Validation of APCI(+) LC-MS/MS Method for the Determination Apricot Byproducts Carotenoids

1.3.1. Fragmentation Pattern of Carotenoids

An oxocarotenoid, trans-β-apo-8′-carotenal, was used as internal standard to counteract potential experimental errors. Identification and quantification of target carotenoids (β-carotene, zeaxanthin and lutein) was conducted by selected-reaction monitoring (SRM) mode. The mass transitions that served as diagnostic *m*/*z* ions for each compound are presented in Table S8. The MS/MS fragment of β-carotene (*m*/*z* = 444.4) match to toluene loss, which is typical in polyene chains. Fragmentation pattern of isomer structures of lutein and zeaxanthin is the same, therefore the different intensities ratios of their product ions is the key for their identification. In the case of lutein, the intensity of product ion with *m*/*z* = 551.4 is higher than that of parent ion of *m*/*z* = 569.4, which, contrarily, is the most abundant fragment in zeaxanthin spectra. The differentiation on fragments intensity ratio is ascribed to (i) the distinct number of molecules' chiral centers (three for lutein and two for zeaxanthin) (ii) the position of -OH in structure rings (ε-ring in lutein and β-ring in zeaxanthin) (iii) the stabilization of the ion formed from the elimination of water (*m*/*z* = 551.4), which is induced the presence of -OH in the allylic position of lutein's ε-ring [47]. In addition, lutein is distinguished from zeaxanthin by fragment ion with *m*/*z* = 495.3, which is observed only during the fragmentation of lutein and is a product of the loss of a water molecule from the protonated parent ion and the retro-Diels-Alder cleavage of a-ionone ring [48]. Likewise, the diagnostic ion of trans-β-apo-8′-carotenal is also the result of the loss of a water molecule (*m*/*z* = 399.3) [47].

1.3.2. Matrix-Matched Calibration Curve and Linearity

Matrix-matched calibration curves were obtained by using pooled mixes of optimal UAE and MAE extracts, respectively. Matrix-matched calibration curves, used for carotenoids quantification,

were constructed by adding five different concentrations (from 0.5 to 20 μg mL^{-1}) of spiked samples in 20 mg of apricot dry residue. In parallel, matrix-free calibration curves (neat samples) of carotenoids standards, at the same concentration range, were also acquired. Internal standard (IS) concentration was 1 μg mL^{-1} in all calibration curves. The linearity of all curves was good with $R^2 \geq 0.9$. The analytical figures of merit of calibration curves are explicitly presented in Table S7. Blank samples (MeOH:MTBE 1:1) were added to sample batch to clean up LC column in order to avoid possible carry-over effects.

1.3.3. Matrix Effect (ME)

Matrix effect (ME) was the indicator of MS signal suppression or enhancement caused to the target carotenoids by co-eluting components. Apricot byproducts is a carotenoid-rich substrate already containing the examined analytes. When the analytes are present in the matrix, ME could be estimated by the ratio of the slope of matrix-matched curve and the slope of neat samples calibration curve. ME calculated with this approach does not dependent on analyte concentration, pre-existing in the extract or added as spiked standard. According to Table S7 a significant signal enhancement (ME(%) ≥ 100) was observed both UAE and MAE carotenoids analysis. Even though APCI is less susceptible to ME than ESI, many researchers observed signal enhancement and not suppression especially when neutral and apolar compounds were analyzed [18].

1.3.4. Limit of Detection (LOD) and Limit of Quantification (LOQ)

Limit of detection (LOD) and limit of quantification (LOQ) were calculated by creating two additional calibration curves (one for UAE and one for MAE) at the area of lowest spiking levels of each carotenoid (Table S7). LODs and LOQs were provided as a result of (3.3sb/a) × IS concentration (μg mL^{-1}) and (10sb/a) × IS concentration (μg mL^{-1}), respectively. In these formulas, a corresponds to calibration curve slope, sb to intercept standard deviation and IS concentration is equal to 1 μg mL^{-1}. In general, LODs/LOQs of β-carotene were higher than those of xanthophylls (Table S7) due to the more intense background noise and matrix interferences at β-carotene elution.

1.3.5. Limit of Detection (LOD) and Limit of Quantification (LOQ)

Method precision was estimated in terms of intra- and inter-day repeatability. Three quality control (QC) samples at three different concentration levels (low, medium, high) were measured to calculate coefficient of variation (CV or RSDr (%)). Intra-day repeatability was carried out by running three replicates of the three QC samples on the same day. Inter-day repeatability was determined from the data obtained by running three replicates of QC samples at three different days. In line with ICH guidelines **[49]**, the developed LC-MS/MS method was precise as RSDr (%) of all QC samples were lower than 15% (Table S8).

Method trueness was expressed as percent relative error (RE%). The limits within a method is considered accurate are 80–110% for concentration levels under 100 μg mL^{-1} and above 100 ng mL^{-1} [18]. RE% was within the limits for all carotenoids standards in both extraction methods (Table S8).

1.3.6. Process Recovery

Process recovery is an indicator of the combined effect of matrix-effect and extraction efficiency. Calculation of process recovery was determined by the equation below:

$$\text{Process recovery(\%)}=[(\text{Peak area of pre-spiked sample-Peak area of analytes in unspiked sample})/\text{Peak area of neat sample}]*100 \quad (3)$$

where pre-spiked sample is the sample with a spiked concentration of standards before the extraction step and neat sample is the sample of the same concentration without the matrix [18]. Although recovery values in the range of 70–120% are generally considered satisfactory, process efficiency is acknowledged as valid when recovery values are (i) precise (ii) reproducible and (iii) over the threshold of 20% [49]. As presented in Table S8, UAE recovery values were almost equal for

β-carotene and lower for zeaxanthin and lutein, compared to MAE. According to Song et al (2015), in a model system, the conversion of trans-lutein to 13-*ci*s-lutein, 13′-*cis*-lutein, 9-*cis*-lutein and 9′-cis lutein follows a second-order kinetics at ~30 °C. Thus the lower UAE recovery of xanthophylls could be assigned to the increased degradation rate under US treatment [24]. Although carotenoids are more susceptible to *cis*-transformation under MW treatment [50,51], in the current work their isomerization seems to be not so pronounced possibly due to significantly lower applied MW power compared to UAE. Overall, outlier recovery values could be ascribed to matrix interferences and lack of suitable isotopically labeled standards.
